# Regulatory circuits involving bud dormancy factor *PpeDAM6*

**DOI:** 10.1038/s41438-021-00706-9

**Published:** 2021-12-01

**Authors:** Alba Lloret, Carles Quesada-Traver, Ana Conejero, Vicent Arbona, Concepción Gómez-Mena, César Petri, Jesús A. Sánchez-Navarro, Elena Zuriaga, Carmen Leida, María Luisa Badenes, Gabino Ríos

**Affiliations:** 1 Instituto Valenciano de Investigaciones Agrarias, 46113 Moncada, Valencia Spain; 2 Departament de Ciències Agràries i del Medi Natural, Universitat Jaume I, Castello de la Plana, Spain; 3 Instituto de Biología Molecular y Celular de Plantas, Universitat Politècnica de València-Consejo Superior de Investigaciones Científicas, 46022 Valencia, Spain; 4 Departamento de Fruticultura Subtropical y Mediterránea, IHSM-UMA-CSIC, Avenida Dr. Wienberg, s/n 29750 Algarrobo-Costa, Málaga Spain

**Keywords:** Agricultural genetics, Transgenic plants, Flowering, Plant physiology

## Abstract

*DORMANCY-ASSOCIATED MADS-BOX* (*DAM*) genes have recently emerged as key potential regulators of the dormancy cycle and climate adaptation in perennial species. Particularly, *PpeDAM6* has been proposed to act as a major repressor of bud dormancy release and bud break in peach (*Prunus persica*). *PpeDAM6* expression is downregulated concomitantly with the perception of a given genotype-dependent accumulation of winter chilling time, and the coincident enrichment in H3K27me3 chromatin modification at a specific genomic region. We have identified three peach BASIC PENTACYSTEINE PROTEINs (PpeBPCs) interacting with two GA-repeat motifs present in this H3K27me3-enriched region. Moreover, PpeBPC1 represses *PpeDAM6* promoter activity by transient expression experiments. On the other hand, the heterologous overexpression of *PpeDAM6* in European plum (*Prunus domestica*) alters plant vegetative growth, resulting in dwarf plants tending toward shoot meristem collapse. These alterations in vegetative growth of transgenic lines associate with impaired hormone homeostasis due to the modulation of genes involved in jasmonic acid, cytokinin, abscisic acid, and gibberellin pathways, and the downregulation of shoot meristem factors, specifically in transgenic leaf and apical tissues. The expression of many of these genes is also modified in flower buds of peach concomitantly with *PpeDAM6* downregulation, which suggests a role of hormone homeostasis mechanisms in *PpeDAM6*-dependent maintenance of floral bud dormancy and growth repression.

## Introduction

Throughout evolution, perennial plants have developed different strategies to adapt to seasonal changing environmental conditions. Among them, dormancy facilitates survival of growing tissues under the low and freezing temperatures of autumn and winter by interrupting cell division and growth, and activating general and specific defense mechanisms^1,2^.

Prior to bud dormancy induction, cessation of meristem growth and bud set are induced by photoperiod changes (short daylength) and/or low temperature conditions in apical vegetative meristems^3,4^, whereas the growth of axillary vegetative meristems and differentiated flowers is stopped by correlative bud inhibition^5^. Paradoxically, bud dormancy completion is also favored by prolonged chilling^6^. This chilling requirement for dormancy release is quantitative and specific for different genotypes. After dormancy release, buds become competent for growing, requiring a period of mild temperatures for initiating bud break. This state is widely known as ecodormancy.

In Rosaceae tree species and other perennial plants, *DORMANCY-ASSOCIATED MADS-BOX* (*DAM*) genes, phylogenetically related to the *Arabidopsis thaliana* flowering factor *SHORT VEGETATIVE PHASE* (*SVP*), act as key regulators of bud dormancy maintenance and release^7–9^. A deletion of four out of six tandemly repeated *DAM* genes has been proposed to cause the non-dormant phenotype of the *evergrowing* (*evg*) mutant of peach (*Prunus persica*)^10,11^. In addition, RNA silencing of the *MdDAM1* gene results in an *evg*-like phenotype in apple^12^. The ectopic expression of *DAM1* gene from leafy spurge (*Euphorbia esula*) delays flowering and decreases the expression of the flowering gene *FLOWERING LOCUS T* (*FT*) in *Arabidopsis thaliana*^13^. Moreover, *PmDAM6* gene from Japanese apricot induces early growth cessation and terminal bud set when overexpressed in transgenic poplar^7^ and apple^9^. Consistently, transgenic plants overexpressing apple *MdoDAMb* and *MdoSVPa* genes show delayed bud break^8^.

The expression of *DAM*-like genes has been found closely associated with the dormancy status of buds in several species^7,14–16^, but few specific regulatory elements and factors have been found to integrate environmental and developmental inputs on *DAM*- like expression so far. Among them, C-REPEAT BINDING FACTOR (CBF)-like factors involved in cold acclimation processes are able to bind *PmDAM6* promoter of Japanese apricot in the yeast one-hybrid (Y1H) system^17^, and activate the promoters of pear *PpDAM1* and *PpMADS13-1* in transient reporter assays^18,19^. On the other hand, ABA dependence of *DAM*-like expression is conferred by the specific binding of ABA-responsive element-binding factors PpAREB1^20^ and PpyABF3^21^, with opposite inhibiting and activating effects on *PpDAM1* and *PpyDAM3* expression, respectively.

A succession of epigenetic-related events has been found associated with *DAM*-like repression and dormancy release in different species, resembling *FLOWERING LOCUS C* (*FLC*) regulation by vernalization in *Arabidopsis thaliana*^22,23^. In peach, *PpeDAM6* downregulation associates with H3K4me3, H3 deacetylation, and H3K27me3 enrichment on specific promoter and intronic regions of the gene^24,25^. Coordinately with histone modifications, other epigenetic-related mechanisms involving accumulation of small RNA and DNA methylation support a prominent role of *PpeDAM4* in controlling floral bud dormancy in peach^11^. A similar decrease in dormancy-dependent H3K4me3 enrichment has been observed in *PavDAM5-6* genes from sweet cherry^26^, and *PpMADS13-1* gene from Japanese pear^18^.

Moreover, *DAM*-like genes have been proposed to regulate hormone biosynthesis genes. Thus, pear *PpDAM1* binds and upregulates the expression of *PpNCED3* gene, coding for a 9-cis-epoxycarotenoid dioxygenase involved in ABA biosynthesis, in close agreement with changes in ABA content across flower bud development^20^.

In this study, we have identified regulatory factors binding the GA-repeat sequences within a region enriched in H3K27me3 in *PpeDAM6* gene in dormancy-released floral buds of peach^25^, and have postulated its participation in dormancy-dependent *PpeDAM6* repression. We have also studied *PpeDAM6* regulatory circuits by expressing ectopically the gene in plum (*Prunus domestica*) transgenic plants.

## Results

### BPC family proteins bind a regulatory intronic region of *PpeDAM6* gene

**Fig. 1:**
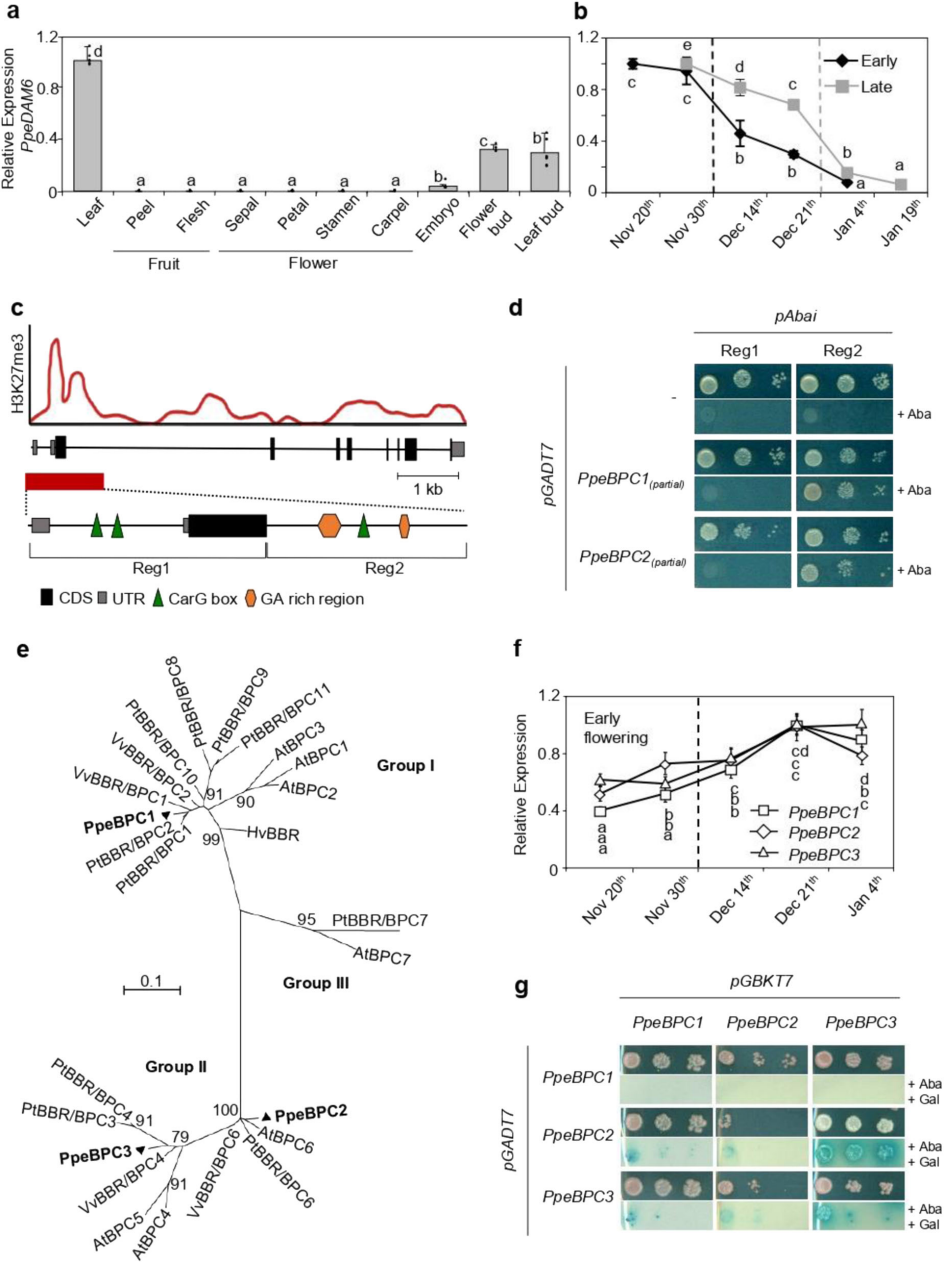
BPC family proteins interact with GA-repeat motifs in *PpeDAM6*. **a**, **b** Relative expression of *PpeDAM6* in peach by real-time RT-PCR. Data are means from three biological samples with two technical replicates each, with error bars representing standard deviation. Different letters (a–e) indicate significant difference between samples with a confidence level of 95% in each cultivar. **a** Different plant tissues. Tubulin-like and actin-like genes were used as reference genes. **b** Floral bud samples from early (black line) and late (gray line) flowering cultivars. Dash lines represent dormancy release for each cultivar. SAND-like gene was used as reference gene. **c** Schematic representation of H3K27me3-enriched region (red rectangle) of *PpeDAM6* adapted from Leida et al.^24^ and the designated baits for Y1H assay (Reg1 and Reg2). Exon organization of *PpeDAM6* (black rectangles) and untranslated 5’ and 3’ regions (gray rectangles), CarG box (green triangle), and GA-repeat motifs (brown pentagons) are shown. **d** Y1H analysis of different combinations of pABAi vectors with Reg1 and Reg2 regions and prey vectors (pGADT7) containing positive screening partial clones of *PpeBPC1* and *PpeBPC2*, and control plasmids (–). Yeast strains were grown on a minimal medium and a growth selective medium containing 200 μM of Aureobasidin A (+AbA). **e** Phylogenetic tree of BPC proteins from *Arabidopsis, Hordeum vulgare, Populus trichocarpa, Vitis vinifera*, and *Prunus persica*. The tree was constructed using the Maximum Likelihood method and bootstrapped with 1000 replicates. The scale bar indicates the branch length that corresponds to the number of substitutions per amino acid position. **f** Relative expression of *PpeBPC1* (white squares), *PpeBPC2* (white rhombs), and *PpeBPC3* (white triangles) measured along floral bud development in the early flowering cultivar. Dash line represents dormancy release. SAND-like gene was used as reference gene. Data are means from three biological samples with two technical replicates each, with error bars representing standard deviation. Different letters (a–d) indicate significant difference between samples for each gene, at a confidence level of 95%. **g** Y2H analysis of protein interactions between different combinations of bait vectors (pGBKT7) and prey vectors (pGADT7), containing *PpeBPC1, PpeBPC2*, and *PpeBPC3*. Yeast strains were grown on a minimal medium (SD without leucine and tryptophan) and a chromogenic medium containing Aureobasidin A and X-α-Gal (+AbA +Gal)

*PpeDAM6* was highly expressed in leaf, flower, and vegetative buds and noticeably less in embryo, whereas its expression was practically imperceptible in fruit and flower components (Fig. 1a). The fact that *PpeDAM6* was appreciably expressed in tissues that display growth arrest and dormancy mechanisms evidences its patent relationship with these processes. We analyzed *PpeDAM6* expression profile along floral bud development in two cultivars with different low (“early”) and medium (“late”) chilling requirements for dormancy release. *PpeDAM6* was timely downregulated in these cultivars according to their different estimated dormancy release dates, when their respective chilling requirements were achieved (Fig. 1b).

A region spanning about 1.1 kb of *PpeDAM6*, containing the first intron, the translation start site, and part of the large second intron of the gene, was found previously enriched in the repressive histone mark H3K27me3 concomitantly with dormancy release^25^ (Fig. 1c). In order to identify putative regulatory factors that specifically bind to this region, we performed a Y1H approach. This region was divided into two fragments of 558 bp (“Reg1”) and 575 bp (“Reg2”) that were used independently as baits against a cDNA expression library made from mixed dormant and dormancy-released flower bud samples. Reg1 and Reg2 included several CArG box elements (CC(A/T)_6_GG motif recognized by MADS-box domain proteins) and two stretches with, respectively, 19 and 9 GA tandem repeats (Fig. 1c and Supplementary Fig. S1). We screened 10^6^ and 5 × 10^5^ yeast transformants with pABAi-Reg1 and pABAi-Reg2 baits, respectively. No positive candidates were obtained in Reg1 screening, whereas two positive clones corresponding to partial sequences of Prupe.1G338500 and Prupe.1G369400 transcripts bound Reg2 fragment containing the start of the second intron of *PpeDAM6* (Fig. 1d and Supplementary Fig. S1). By BLASTP analysis against “Peach v2.1” genome database^27^, we detected an additional peach transcript highly similar to Y1H positive sequences (Prupe.8G082900). The deduced proteins of these genes contain a GA-repeat binding domain, which has been previously described in the BARLEY B RECOMBINANT (BBR)/BASIC PENTACYSTEINE PROTEIN (BPC) protein family. Thus, from now on we will use the names *PpeBPC1*, *PpeBPC2*, and *PpeBPC3* to designate Prupe.1G338500, Prupe.1G369400, and Prupe.8G082900 genes, respectively.

A phylogenetic tree was constructed using protein sequences of previously characterized *BPC* genes from *Arabidopsis thaliana*, *Hordeum vulgare*, *Populus trichocarpa*, and *Vitis vinifera*^28,29^. As shown in Fig. 1e, BPC proteins clustered into three groups (I, II, and III), in agreement with previous studies^30^. PpeBPC1 fell into group I, while PpeBPC2 and PpeBPC3 were part of group II. Within group II, PpeBPC2 clustered with AtBPC6, PtBBR/BPC6, and VvBBR/BPC6, suggesting that PpeBPC2 could structurally and functionally resemble BPC6-like proteins. *PpeBPC1*, *PpeBPC2*, and *PpeBPC3* gene expression profiles were very similar, showing a slight increase along flower bud development in both early and late flowering cultivars, unlinked to dormancy release dates (Fig. 1f, showing early flowering cultivar data).

By yeast two-hybrid system (Y2H), we confirmed that PpeBPC proteins are potentially able to form heterodimers with each other, as stated in other species (Fig. 1g). However, no interaction was observed with other elements of repressive complexes described as BPC interactors in previous reports, such as the peach orthologs of LIKE HETEROCHROMATIN PROTEIN1 (LHP1), SWINGER (SWN), and SEUSS (Supplementary Fig. S2).

### PpeBPC1 represses *PpeDAM6* by binding to GA-repeat motifs

**Fig. 2:**
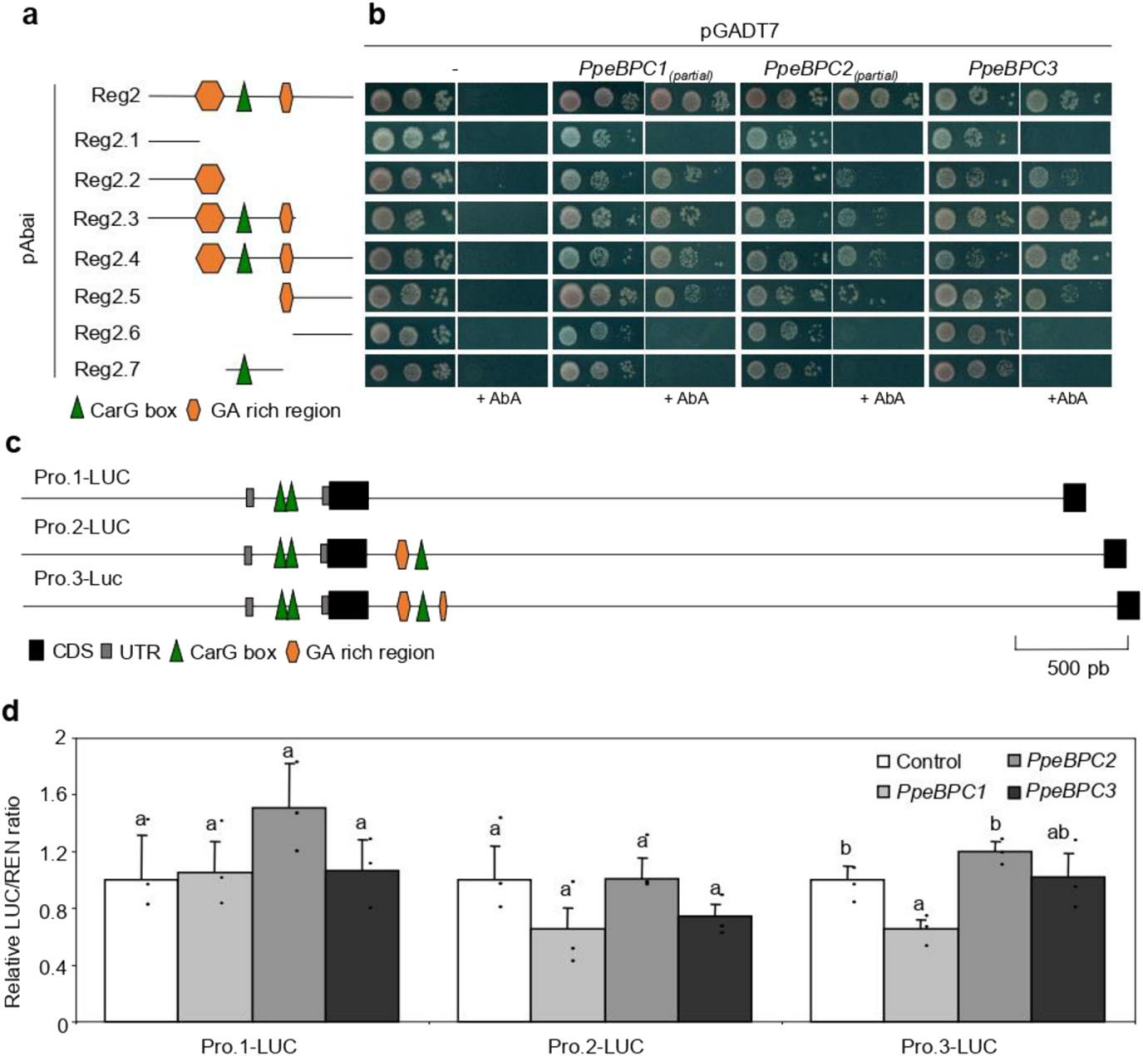
PpeBPC1 represses *PpeDAM6* by binding to GA-repeat motifs in H3K27me3-enriched region. **a** Schematic representation of the designated baits to determine the DNA-binding specificity of peach BPC factors. The positive bait (Reg2) was split in seven different fragments. Potential binding sites like CarG boxes and GA-repeat motifs are labeled with green triangles and brown pentagons, respectively. **b** Y1H analysis of different combinations of pABAi vectors with the seven different regulatory fragments, and prey vectors (pGADT7) with *PpeBPC1*, *PpeBPC2*, and *PpBPC3* and control plasmid (–). Yeast strains were grown on a minimal medium and a growth selective medium containing 200 μM of Aureobasidin A (+AbA). **c** Schematic representation of the different reporter vector constructions for the dual luciferase assay. A genomic fragment including promoter (1 kb), 5’ untranslated region (5’-UTR) (gray rectangles), and first and second exons (black rectangles) is represented. Potential binding sites like CarG boxes and GA-repeat motifs are labeled by green triangles and brown pentagons, respectively. Different reporter constructions show deletions of one or both GA-repeat motifs. **d** Relative LUC/REN ratio measured in the different combinations of reporter vectors (Pro.1-LUC, Pro.2-LUC, and Pro.3-LUC) and effectors vectors containing control plasmid (white bar), *PpeBPC1* (light gray bar), *PpeBPC2* (dark gray bar), and *PpeBPC3* (black bar). In each combination, the value for reporter construction with empty pGreenII-62sk plasmid (control, white bar) was set to 1. Data are means of three biological replicates with error bars representing standard deviation. Different letters (a–b) indicate significant difference between samples for each reporter construction, at a confidence level of 95%

In order to determine the DNA-binding specificity of peach BPC factors, we used Y1H strains containing reporter constructs with serial deletions in the Reg2 fragment (Fig. 2a). As shown in Fig. 2b, PpeBPC1, PpeBPC2, and PpeBPC3 only activated reporter with constructs containing at least one of the two GA-repeat motifs found in Reg2, indicating that their interaction with the H3K27me3-enriched region of *PpeDAM6* is exclusively mediated by these motifs.

For the purpose of clarifying the role of PpeBPC proteins in *PpeDAM6* gene expression regulation, a dual luciferase transient expression assay was performed in *Nicotiana benthamiana* leaves. We designed effector vectors using the complete coding sequences of *PpeBPCs*. For constructing reporter vectors with the luciferase gene (*LUC*) we cloned a *PpeDAM6* genomic fragment including the promoter (1 kb), 5’ untranslated region, translation start site, and full first and second introns (Fig. 2c). Three different versions of this vector containing none (Pro.1-LUC), one (Pro.2-LUC), and two GA-repeat motifs (Pro.3-LUC) were used (Fig. 2c). A second reporter expressing the *Renilla* luciferase gene (REN) under 35S promoter was employed as an internal reference. According to dual luciferase results, there was a slight reduction of LUC/REN ratio when *PpeBPC1* was co-infiltrated with Pro.3-LUC vector, suggesting that GA-repeat motifs are necessary for the interaction between the PpeBPC1 protein and the *PpeDAM6* regulatory region, and PpeBPC1 could act as a transcriptional repressor of *PpeDAM6* (Fig. 2d).

### *PpeDAM6* overexpression impairs growth in plum

**Fig. 3:**
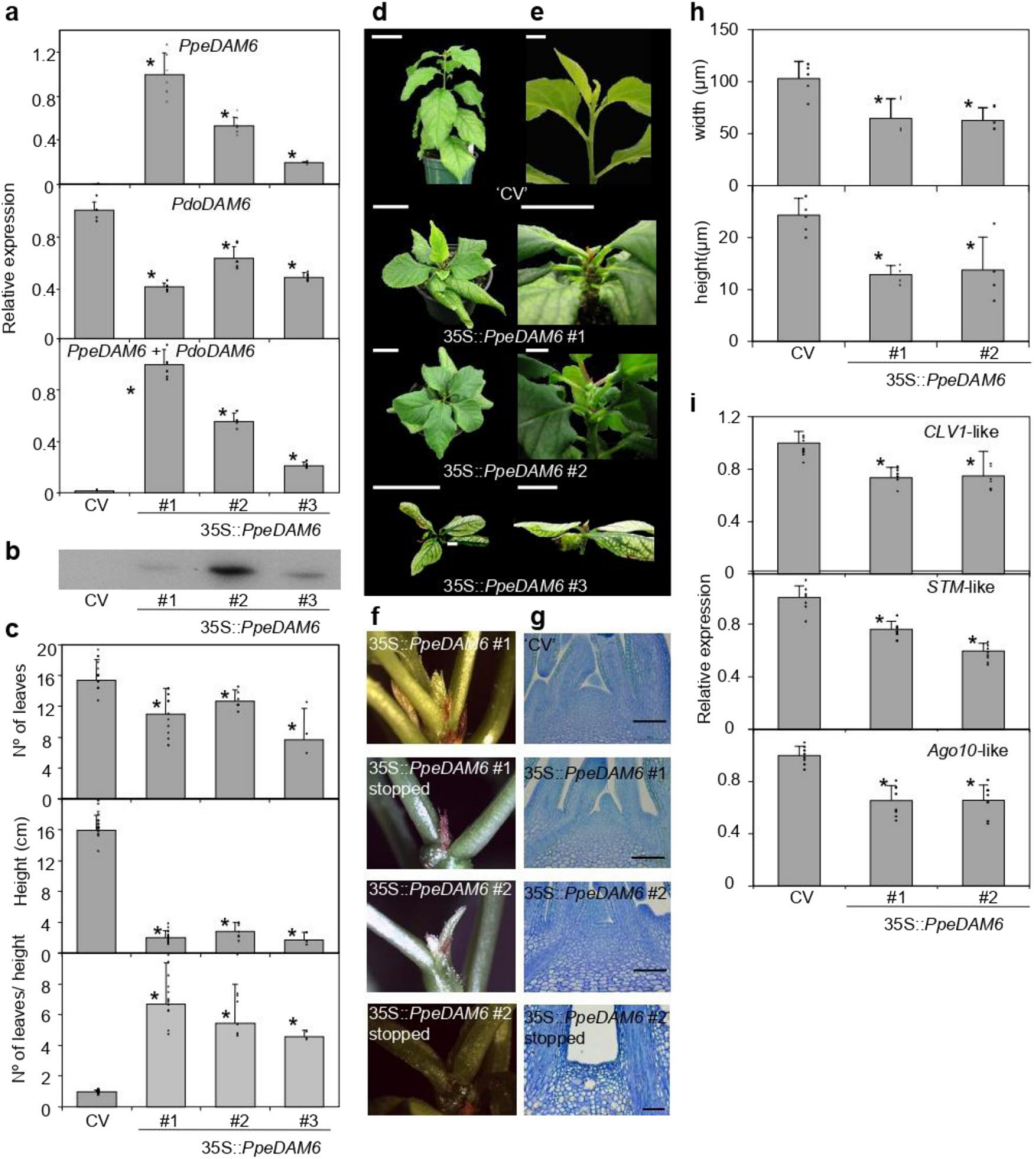
*PpeDAM6* overexpression impairs growth in plum through shoot apical meristem development. **a** Relative expression of heterologous *PpeDAM6*, *PdoDAM6*, and both genes (*PpeDAM6* + *PdoDAM6*) in leaves of three transgenic lines. *AGL26*-like and *actin*-like genes were used as reference genes. Data are means from three biological samples with two technical replicates each, with error bars representing standard deviation. **b** Protein level of PpeDAM6 in leaves of “Claudia Verde” (CV) and transgenic lines 35S::*PpeDAM6* #1, #2, and #3. **c** Different whole plant parameters of 3-month old plants. Data are means from at least three different plants per genotype, with error bars representing standard deviation. An asterisk indicates significant difference with the control at a confidence level of 95%. **d** Phenotype of three-month old plants of CV and transgenic lines. Scale bar, 5 cm. **e** Photographic details of shoot apex. Scale bar, 1 cm. **f** Shoot apex phenotype of transgenic lines 35S::*PpeDAM6* #1 and #2 before and after growth cessation and meristem collapse. **g** Longitudinal section of shoot apical meristem of “Claudia Verde” (CV) and transgenic lines 35S::*PpeDAM6* #1 and #2. Scale bars, 50 µm. **h** Shoot apical meristem width and height in CV and transgenic lines 35S::*PpeDAM6* #1 and #2. Values shown are mean from at least four different plants per genotype with error bars representing standard deviation. **i** Relative expression of *CLV1-*like, *STM*-like, and *AGO10*-like in CV and 35S::PpeDAM6 #1, #2, and #3 apices. *AGL26*-like and *actin*-like genes were used as reference genes. Data are means from four biological apices with two technical replicates each, with error bars representing standard deviation. An asterisk indicates significant difference with the control at a confidence level of 95%

We transformed European plum (*Prunus domestica* cv. “Claudia Verde,” “CV”) with the constitutive expression vector producing PpeDAM6 with c-myc epitope in its N-terminal end. Since current transformation protocols show low efficiency in peach, European plum offers some advantages over other species for functional studies: its taxonomical proximity to peach and similar bud dormancy behavior^31^. After transformation, three independent plum lines expressing 35S::*PpeDAM6* in leaves were identified by quantitative real-time RT-PCR (qRT-PCR). In the three lines, *PpeDAM6* was highly expressed and contributed to most of the combined expression of *DAM6* genes from both species (*PpeDAM6* + *PdoDAM6*) (Fig. 3a). On the other hand, the expression of plum Pdo*DAM6* was slightly reduced in transgenic lines compared with the control “CV.” The presence of PpeDAM6 protein was detected by western blot analysis (Fig. 3b). The results showed poor correlation between mRNA and protein expression levels and protein accumulation, since leaves from line #1 showed higher *PpeDAM6* transcript expression by qRT-PCR, whereas protein accumulation was higher in line #2 (Fig. 3a, b).

Transgenic lines exhibited drastic alterations in vegetative development. 35S::*PpeDAM6* transformed plants were shorter, despite the fact that they developed about the same number of leaves than the control (Fig. 3c). Consequently, internodes were shorter (Fig. 3d). CV was not a true control since the genetic background of transformants differs due to seed segregation of heterozygous parents. However, these alterations were present in the three *PpeDAM6* transformants and absent in the different lines produced in the same in vitro procedure with control plasmid and also without plasmidic DNA (CV lines), arguing for a transgene-dependent effect. Unfortunately, most transgenic shoot apices ceased growth few months after plant acclimatization (Fig. 3e, f). Microscopic sections showed a total extinction of the shoot apical meristem (SAM) in plants that ceased growth (Fig. 3g), and reduced SAM dimensions (width and height) in actively growing 35S::*PpeDAM6* plants (Fig. 3h). Excised apices of 35S::*PpeDAM6* plants showed a concomitant downregulation of SAM development and the organization genes *CLAVATA1* (*CLV1*)-like, *SHOOT MERISTEMLESS* (*STM*)-like, and *ARGONAUTE10* (*AGO10*)-like (Fig. 3i). These alterations in meristem proliferation precluded any attempt to obtain reproductively competent 35S::*PpeDAM6* plants, and consequently no direct functional evidences about the role of *PpeDAM6* on floral bud dormancy could be obtained. In this point, considering that the native expression of *PpeDAM6* genes is not constrained to dormant organs and in fact it is highly expressed in leaves (Fig. 1a), we decided to continue the analysis of transgenic leaves and apices in order to achieve general mechanistic clues about the molecular activity of *PpeDAM6* in this heterologous model, to be subsequently tested by expression studies in dormant tissues. However, only functional approaches performed in flower buds could confirm the relevance of these mechanisms in the dormancy process.

We analyzed the global expression pattern of leaves from 3-month-old 35S::*PpeDAM6* transgenic plum lines #1 and #2 and control “CV” by RNA-seq analysis (three replicates per sample). The transcriptomic sequences were uploaded to NCBI BioProject database (ID PRJNA630876). High-throughput sequencing resulted in 84 million high-quality paired-end reads per replicate (Supplementary Table S1). Clean reads were successfully de novo assembled by Trinity, leading to the identification of 187,901 unigenes (Supplementary Table S2).

The overexpression of *PpeDAM6* modified the expression of around 13,000 differentially expressed unigenes (DEUs) in both transgenic lines #1 and #2, from which 6494 were upregulated and 6640 were downregulated in 35S::*PpeDAM6* plants (Supplementary Fig. S3a). Eleven Kyoto Encyclopedia of Genes and Genomes (KEGG) pathways were significantly upregulated in both lines, whereas 14 were downregulated, among which “ribosome” (ko03010) and “carbon metabolism” (map01200) accounted for the largest proportion of DEUs (Supplementary Fig. S3b, c). Several essential pathways for plant survival and development were downregulated in both transformed lines, such as “photosynthesis-antenna pathway” (map00196), “photosynthesis” (ko00195), “nitrogen metabolism” (map00910), and “carbon fixation in photosynthetic organisms” (ko00710). The analysis of KEGG pathways suggested that *PpeDAM6* overexpressing plum lines had lower cellular activity, in agreement with their dwarf phenotype. KEGG enrichment analysis also revealed that “alpha-linolenic acid metabolism” (map00592), involved in jasmonic acid (JA) biosynthesis, was significantly upregulated in 35S::*PpeDAM6* transgenic plum, whereas “plant hormone signal transduction” (map04075) was downregulated (Supplementary Fig. S3b, c).

### *PpeDAM6* overexpression modifies hormones synthesis and response

Subsequently, we evaluated the contribution of hormone-related pathways to the transcriptome of 35S::*PpeDAM6* transgenic plants. We found DEUs associated with various aspects of hormone homeostasis and response, mostly related to ABA, cytokinin (CK), GA, and JA hormones (Supplementary Table S3).

**Fig. 4:**
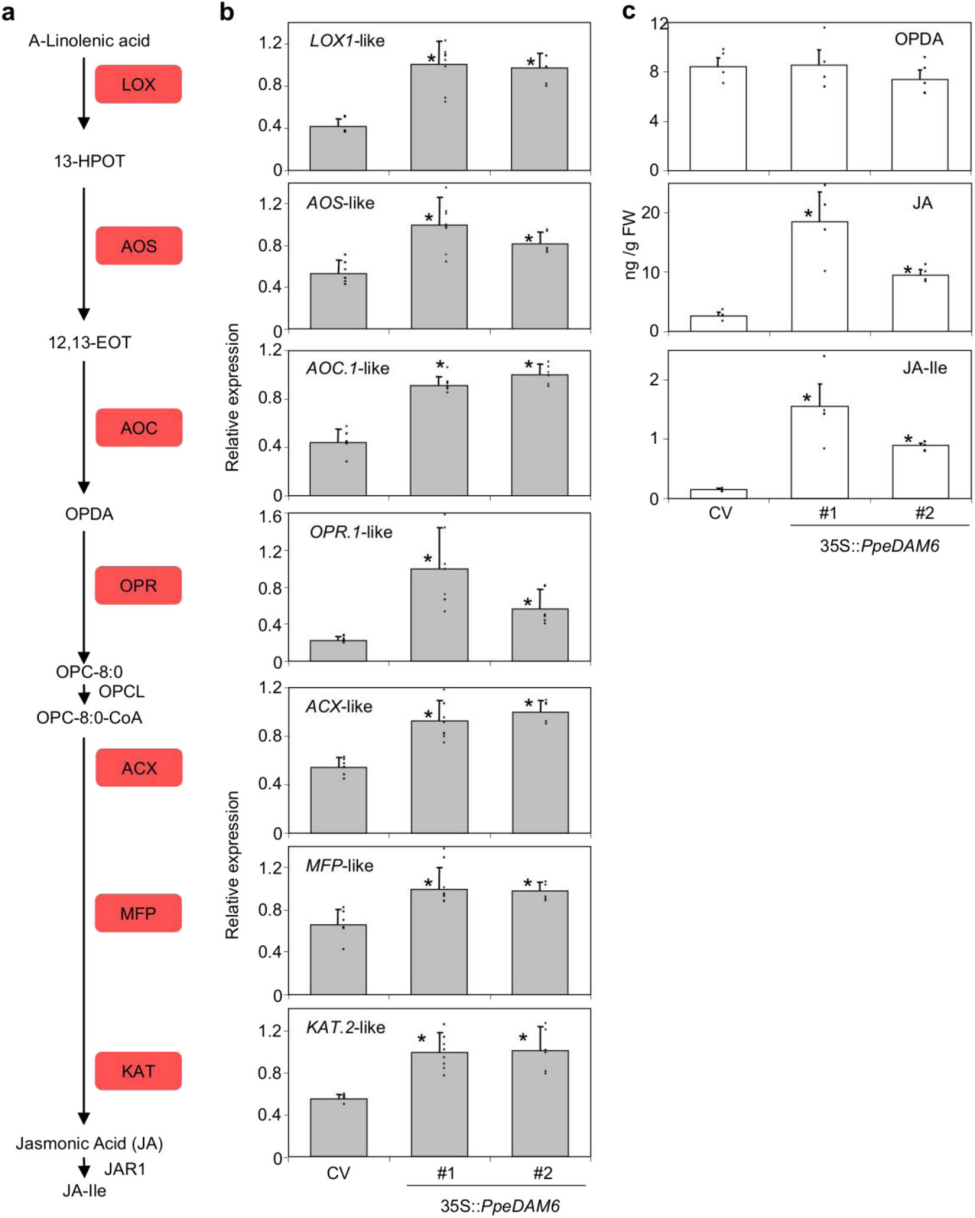
JA biosynthesis pathway in 35S::*PpeDAM6* overexpressing lines. **a** Simplified overview of JA biosynthesis pathway. **b** Relative expression levels of JA biosynthesis genes in leaves of Claudia Verde (CV) and 35S::*PpeDAM6* #1 and #2. *AGL26*-like and *actin*-like genes were used as reference genes. Data are means from three biological samples with two technical replicates each, with error bars representing standard deviation. **c** OPDA, JA, and JA-Ile content in leaves of CV and 35S::*PpeDAM6* #1 and #2. Data are means from four biological samples, with error bars representing standard deviation. An asterisk indicates significant difference with the control at a confidence level of 95%

The JA biosynthetic genes were found upregulated in both transgenic lines, from *13-LYPOXIGENASE1*-like (*LOX1*-like) to *3-KETOACYL-COA THIOLASE*-like (*KAT2*-like), with the exception of *OPC-8:0 COA LIGASE* (*OPCL*) (Fig. 4a, b). Such enhanced expression level of JA biosynthetic genes correlated well with JA and (+)-7-*iso-*JA-Ile (JA-Ile) hormone content, but we found no difference in the content of the precursor *cis-*(+)-12-oxo-phytodienoic acid (OPDA) (Fig. 4c).

**Fig. 5:**
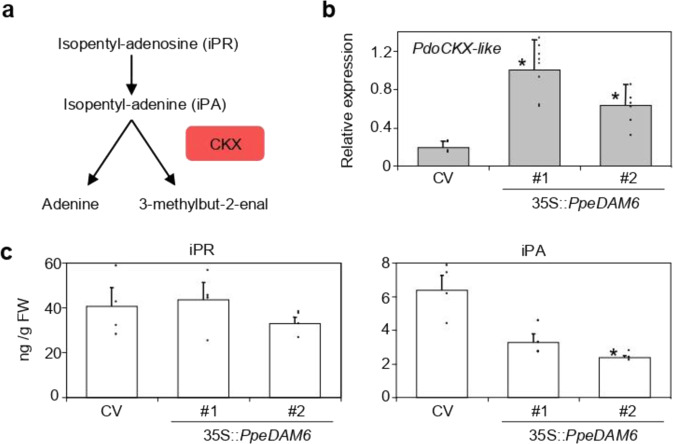
CK biosynthesis pathway in 35S::*PpeDAM6* overexpressing lines. **a** Simplified overview of CK catabolism pathway. **b** Relative expression levels of CKX genes in leaves of Claudia Verde (CV) and 35S::*PpeDAM6* #1 and #2. *AGL26*-like and *actin*-like genes were used as reference genes. Data are means from three biological samples with two technical replicates each, with error bars representing standard deviation. **c** Content of iPR and iPA in leaves of CV and 35S::*PpeDAM6* #1 and #2. Data are means from four biological samples, with error bars representing standard deviation. An asterisk indicates significant difference with the control at a confidence level of 95%

The expression of *CYTOKININ DEHYDROGENASE*-like gene (*CKX*-like), which catalyzes the irreversible degradation of CKs and is thus a key regulator of CK content in plants (Fig. 5a), was highly increased by *PpeDAM6* overexpression (Fig. 5b). In close agreement with these results, the content of the CK hormone isopentyl-adenine (iPA) was reduced in leaves of transformed plum plants compared with wild-type “CV” (Fig. 5c).

**Fig. 6:**
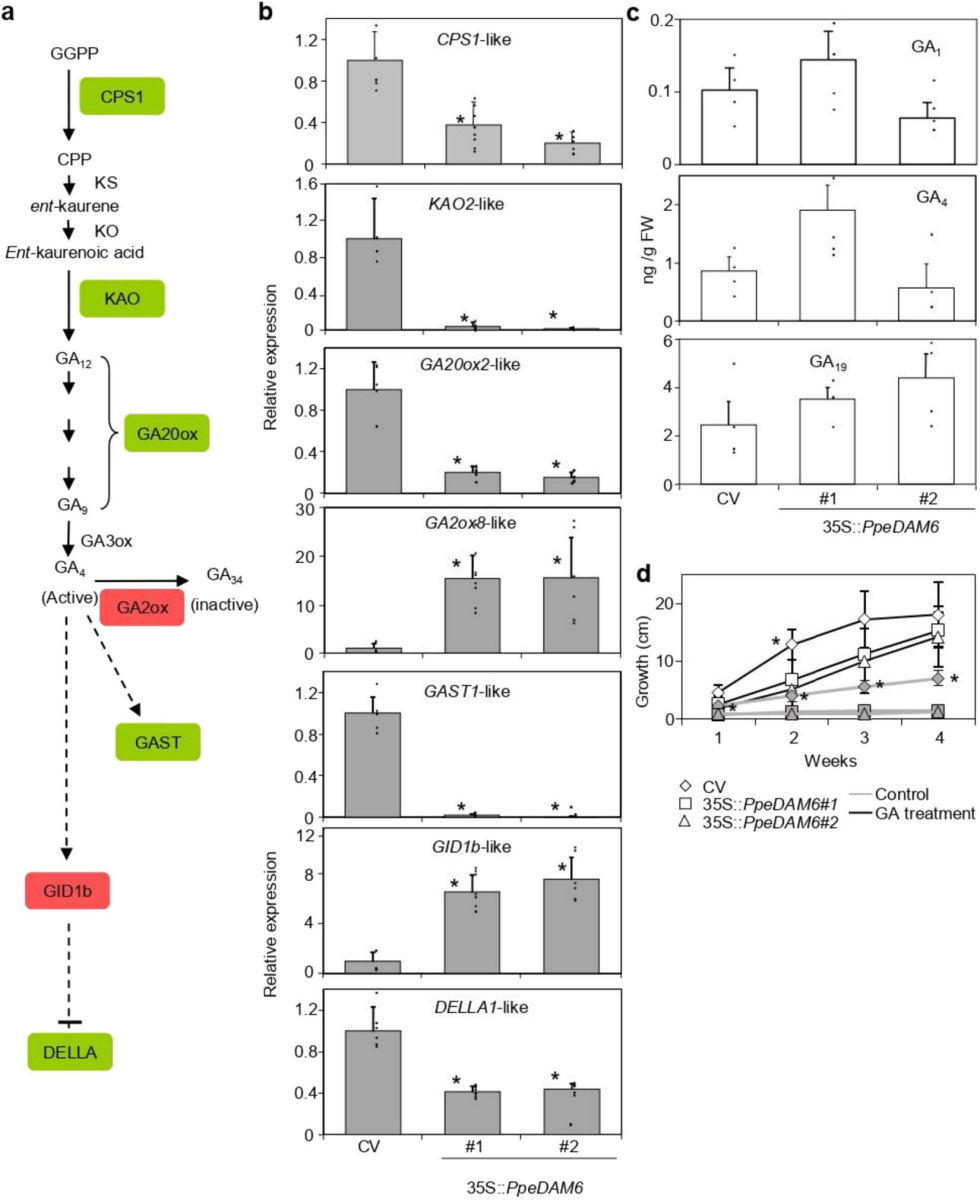
GA biosynthesis and response pathways in 35S::*PpeDAM6* overexpressing lines. **a** Simplified overview of GA biosynthesis and signaling pathway. **b** Relative expression levels of GA-related genes in leaves of Claudia Verde (CV) and 35S::*PpeDAM6* #1 and #2. *AGL26*-like and *actin*-like genes were used as reference genes. Data are means from three biological samples with two technical replicates each, with error bars representing standard deviation. An asterisk indicates significant difference with the control at a confidence level of 95%. **c** Content of GA19 and GA4 in leaves of CV and 35S::*PpeDAM6* #1 and #2. Data are means from four biological samples, with error bars representing standard deviation. **d** Growth of CV (white rhombs), 35S::*PpeDAM6* #1 (white squares) and #2 (white triangle) under water (control) and GA treatments. Data are means from at least three different plants per genotype. Different letters (a–b) indicate significant difference between different genotypes in each week, at a confidence level of 95%

Likewise, genes involved in GA biosynthesis, catabolism, and signal transduction pathways were identified (Fig. 6a). In GA biosynthetic pathway, *ENT-COPALYL DIPHOSPHATE SYNTHASE 1*-like (*CPS1*-like), *ENT-KAURENOIC ACID OXIDASE 2*-like (*KAO2*-like), and *GA20-OXIDASE 2*-like (*GA20OX2*-like) were downregulated, while the GA catabolic gene *GA2-OXIDASE 8*-like (*GA2OX8*-like) was upregulated in transgenic lines. With respect to GA signaling pathway, we found the GA receptor *GIBBERELLIN INSENSITIVE DWARF1b*-like (*GID1b*-like) upregulated, while *GA-STIMULATED TRANSCRIPT 1*-like (*GAST1*-like) and the GA signaling repressor *DELLA1*-like were downregulated (Fig. 6b). Despite the fact that gene expression analysis in the GA pathway suggested a reduction of bioactive GA content in transformed plum plants, we could not detect consistent changes in three GAs accumulated at detectable levels (GA_1_, GA_4_, and GA_19_) (Fig. 6c). However, the exogenous application of active GA_3_ significantly enhanced growth of both transgenic lines, becoming similar to the control “CV” (Fig. 6d).

**Fig. 7:**
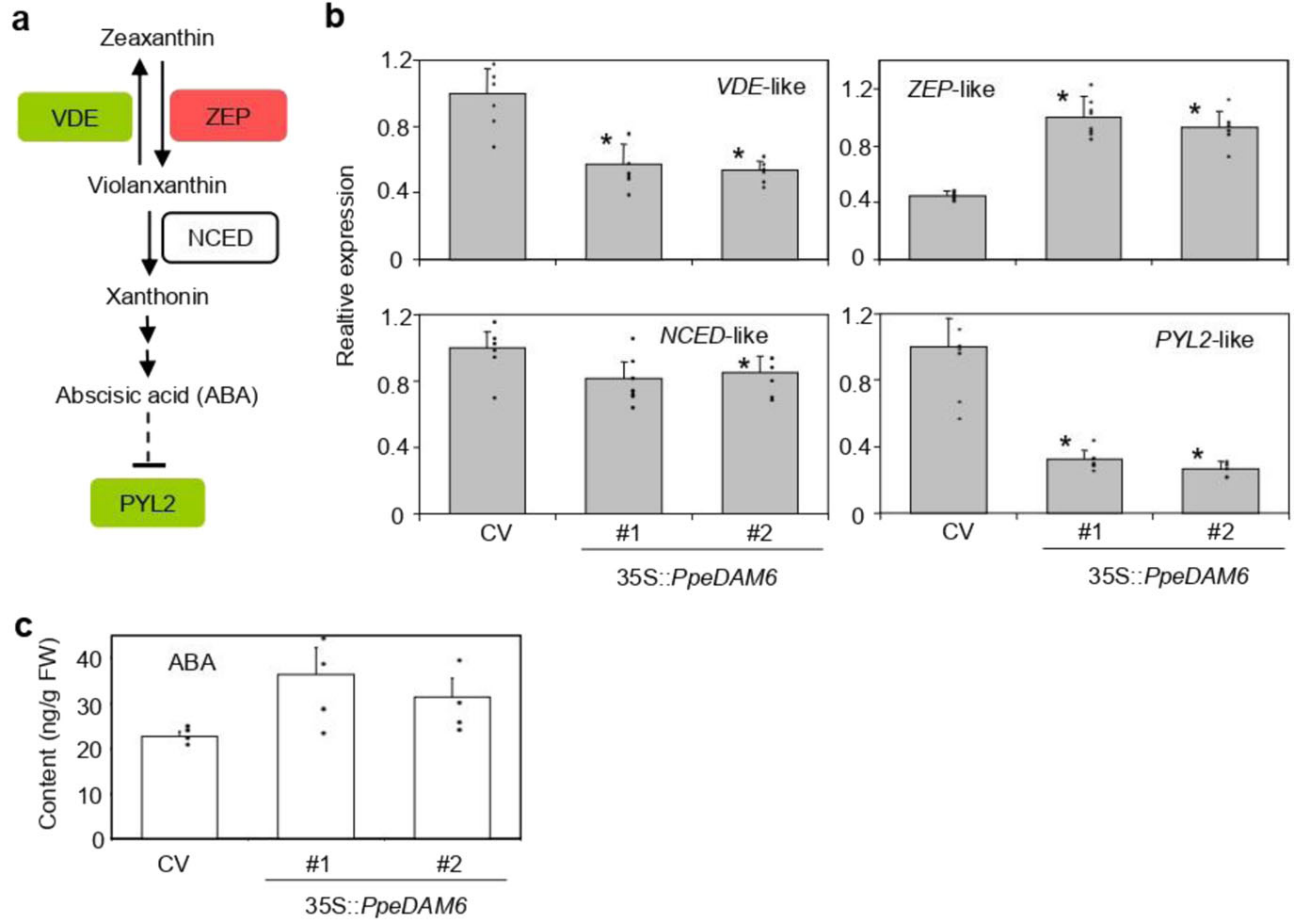
ABA biosynthesis and response pathway in 35S::*PpeDAM6* overexpressing lines. **a** Simplified overview of ABA biosynthesis and signaling pathways. **b** Relative expression levels of ABA-related genes in leaves of Claudia Verde (CV) and 35S::*PpeDAM6* #1 and #2. *AGL26*-like and *actin*-like genes were used as reference genes. Data are means from three biological samples with two technical replicates each, with error bars representing standard deviation. **c** Content of ABA in leaves of CV and 35S::*PpeDAM6* #1 and #2. Data are means from four biological samples, with error bars representing standard deviation. An asterisk indicates significant difference with the control at a confidence level of 95%

Within ABA biosynthesis pathway, the genes *ZEP*-like and *VED*-like encoding zeaxanthin epoxidase and violaxanthin de-epoxidase enzymes are involved in the production of violaxanthin from zeaxanthin and the reverse conversion, respectively (Fig. 7a). In 35S::*PpeDAM6* plants, *ZEP*-like and *VED*-like were respectively up- and downregulated compared to CV (Fig. 7b), promoting the violaxanthin production step. However, the expression of a *NCED*-like gene, codifying for 9-cis-epoxycarotenoid dioxygenase was not significantly altered. Consistently with these data, ABA was over-accumulated in 35S::*PpeDAM6* leaves (Fig. 7c). Interestingly, the ABA receptor gene *PYL2*-like was strongly repressed in *PpeDAM6* overexpressing plants (Fig. 7b), suggesting a complex effect on ABA synthesis and response.

### Hormone accumulation and gene expression in dormant floral buds of peach

The aforementioned genes and pathways were described in the overexpressing heterologous model of transgenic plum by analyzing transgenic leaves and apices. In spite of the growing body of knowledge about the expression and role of *DAM*-like genes in leaves and other vegetative tissues (Fig. 1a), since the purpose of this study focuses on the involvement of *PpeDAM6* in flower bud dormancy promotion, we analyzed hormone accumulation and gene expression in flower buds of peach, a well-known model. Two cultivars with different flowering time behavior were assayed.

**Fig. 8:**
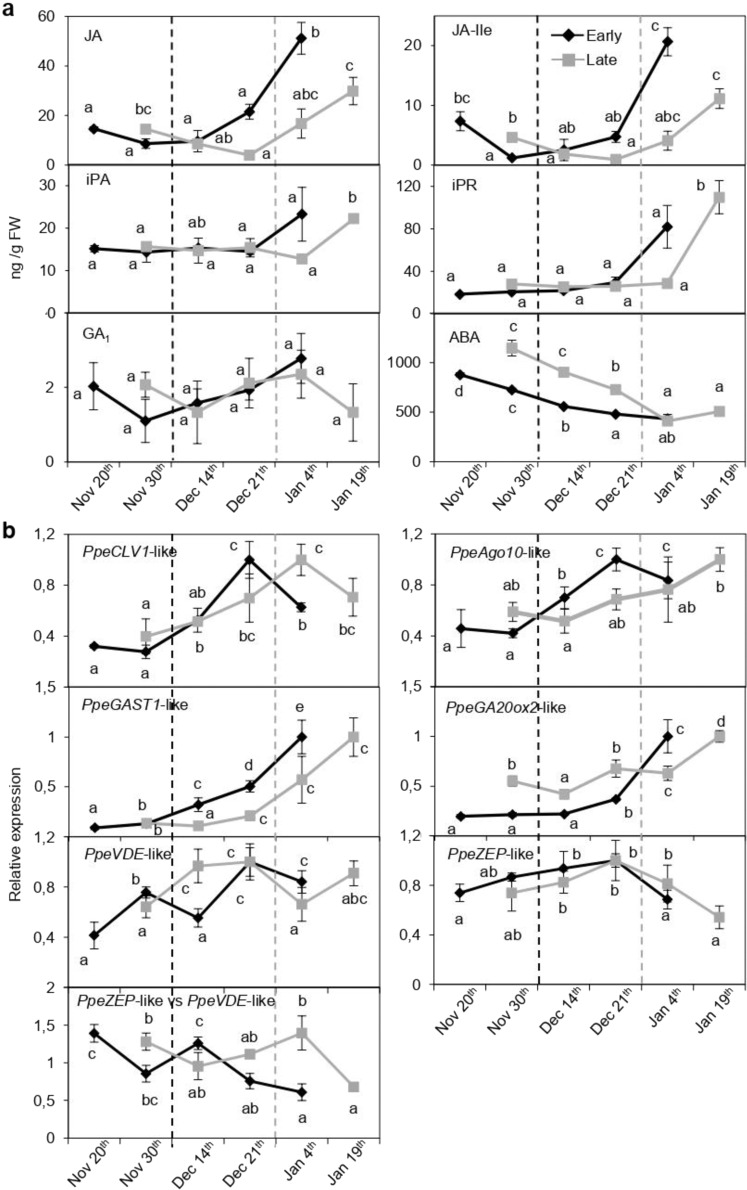
Hormone homeostasis and meristem-related genes during floral bud development in peach. **a** Seasonal changes in the hormone content along floral bud development in early (black line) and late (gray line) flowering cultivars. Dash lines represent dormancy release. Data are means from three biological samples with two technical replicates each, with error bars representing standard deviation. **b** Relative expression of *PpeCLV1-*like, *PpeAGO10-*like*, PpeGAST1-*like, and *PpeGa20ox2*-like measured along floral bud development in early (black line) and late (gray line) flowering cultivars. *SAND*-like gene was used as reference gene. Data are means from three biological samples with two technical replicates each, with error bars representing standard deviation. Different letters (a–e) indicate significant difference between samples, at a confidence level of 95%

The hormones JA and JA-Ile decreased in floral buds of the late flowering cultivar during the progression of dormancy until dormancy release (first three samples), and also JA-Ile in the early flowering cultivar (Fig. 8a), in accordance with *PpeDAM6* downregulation (Fig. 1b). This was in agreement with a higher JA and JA-Ile accumulation observed in leaves of overexpressing *PpeDAM6* plum lines (Fig. 4c). However, after this initial drop, JA and JA-Ile levels sharply increased (Fig. 8a), in parallel to known flowering developmental processes occurring during the ecodormancy stage^32^. The expression analysis of JA biosynthetic genes matched these observations, since *AOS*-like, *AOC1*-like, and *KAT2*-like reduced significantly their expression in the late genotype prior to dormancy release, and *LOX1*-like, *AOS*-like, *OPR1*-like, *OPR2*-like, *ACX*-like, and *MFP*-like were noticeably upregulated after dormancy release (Supplementary Fig. S4).

On the other side, CK levels were coincidently lower in 35S::*PpeDAM6* lines and dormant floral buds, in close agreement with their high *PpeDAM6* levels (Fig. 8a). However, the late and sharp accumulation of iPR and iPA in ecodormant floral bud samples was not associated with an increase in the CK catabolizing gene *CKX*-like in floral buds (Supplementary Fig. S4), which argued for the presence of additional mechanisms for the drastic CK overproduction in floral buds prior to bud break.

GA_1_ level was not changing significantly during floral bud dormancy, despite the fact that GA biosynthesis gene *GA20ox2*-like and GA-response gene *GAST1*-like were upregulated concomitantly with dormancy release and ecodormancy progression (Fig. 8b).

Regarding ABA content, the decreasing hormone level during floral bud development, reaching its lowest stable value after dormancy release in a cultivar-dependent manner (Fig. 8a), consistently matched observations obtained in 35S::*PpeDAM6* plants and previous data reported by the literature. The ratio of *ZEP*-like to *VED*-like gene expression fairly confirmed that the conversion of zeaxanthin to violaxanthin was also a target of ABA synthesis regulation in floral buds (Fig. 8b), whereas *PpeNCED*-like expression did not match ABA levels in this tissue (Supplementary Fig. S4).

On the other side, SAM-related *CLV1*-like and *AGO10*-like genes showed lower expression values in dormant floral buds where *PpeDAM6* was highly expressed, reinforcing the idea that *PpeDAM6* affects *CLV1*-like and *AGO10*-like regulation in the frame of both flower bud and apical meristem developmental switches.

Most importantly, observed variations in hormone and gene expression values were in every case correspondingly earlier in the early flowering cultivar, confirming that they were dependent on the dormancy stage of floral buds, instead of temperature and other environmental inputs.

## Discussion

### BPC proteins bind and regulate *PpeDAM6* expression

In plants, GA-repeat motifs are mainly recognized by a specific family of transcription factors called BBR/BPC, firstly characterized in barley^28^ and subsequently in *Arabidopsis*^30^ and cucumber^33^. Although further research is needed, this study suggests that PpeBPC1 represses *PpeDAM6* transcriptional activity by binding to two of these motifs, located in an intronic region of *PpeDAM6* that becomes enriched in H3K27me3 modification concomitantly with dormancy release events^25^. An association between BPC binding and H3K27me3 enrichment has been already observed in *Arabidopsis*^34^. BBR/BPC family has been related to transcription inhibition via induction of conformational changes in DNA structure, by interacting with themselves or recruiting the repressor SEUSS^35^ and components of the Polycomb Repressive Complex (PRC), such as LIKE HETEROCHROMATIN PROTEIN1 (LHP1)^34^. In addition, BPCs from *Arabidopsis* have been found to downregulate *ABI4* gene by recruiting the PRC2 complex component SWINGER (SWN) to *ABI4* promoter, mediated by the specific H3K27me3 modification^33^. In our Y2H assay, peach BPC proteins interacted with each other, confirming their ability to form homo and heterodimers, but no interaction was found with the respective peach orthologs of LHP1, SWN, and SEUSS, contrarily to findings reported in *Arabidopsis*^33–35^. However, the convergence of PpeBPC binding and H3K27me3 modification in a short regulatory region of *PpeDAM6* suggests that PRC2 complexes and PpeBPC could interact in the dormancy-dependent regulation of *PpeDAM6*.

### *PpeDAM6* as a dedicated growth modulator

Despite *DAM* genes being commonly associated with dormancy establishment and maintenance in many woody species, recent functional insights point to a more general role in growth regulation^36^. In this work, transgenic plums overexpressing *PpeDAM6* show a strong stunted growth that mainly affected internode elongation, in concordance with the altered phenotypes observed in 35S::*PmDAM6* transgenic poplar^7^ and apple^9^. The bud dormancy phenotype of adult plum plants was not analyzed because transgenic lines died few months after soil acclimatization. However, since *PpeDAM6* gene is naturally expressed in leaves of peach, the study of *PpeDAM6* overexpression in transgenic seedlings could provide clues about its overall mode of action, independently of its tissue-specific expression. Our data suggest that *PpeDAM6* affects growth in transgenic lines most likely due to an altered homeostasis of the hormones JA, CK, GA, and ABA, in close agreement with a previous work showing that *PmDAM6* from Japanese apricot decreases CK and increases ABA content in transgenic apple plants^9^.

35S::*PpeDAM6* plants presented higher levels of JA and JA-Ile, due to the upregulation of JA biosynthesis pathway at several steps. This was in agreement with a noticeably higher amount of these hormones in dormant floral buds, to decrease around the dormancy release date. This observation fits well with the previously described growth repressing and cold acclimation promoting activities of JA and its conjugate forms^37^. JA has been described as a key compound conferring abiotic stress tolerance in plants^38^, and particularly affecting cold stress responses through the repressive binding of JASMONATE ZIM-DOMAIN proteins to INDUCER OF CBF EXPRESSION, released by JA^39^. Thus, the activation of JA responses during the dormancy period most likely contributes to improve the tolerance of floral buds to cold and freezing stresses.

Interestingly, after dormancy release drop, both JA and JA-Ile contents increase sharply in both early and late flowering cultivars, coincidently with the beginning of important flowering steps to be accomplished during ecodormancy, such as microsporogenesis and pollen development and maturation^40^. Since JA production and signaling is essential for proper anther filament elongation, pollen viability, and dehiscence of anthers^32^, such burst of JA accumulation may be involved in these processes. On the other hand, a JA increase has been also reported in leaf buds of beech trees^41^ and potato tubers undergoing active sprouting^42^, suggesting that JA production may accompany these bud-break events. The expression profile of biosynthetic genes *LOX1*-like, *AOS*-like, *AOC1*-like, *OPR1*-like, *OPR2*-like, *ACX*-like, *MFP*-like, and *KAT2*-like fits well with reported development and genotype-dependent variations in JA and JA-Ile levels, which highlights the relevance of phenological inputs on the regulation of JA synthesis.

Transgenic 35S::*PpeDAM6* plants show a lower content of the CK iPA, which is consistent with an increased expression level of the catabolic CK oxidase/dehydrogenase (*PdoCKX*-like). In general, CKs affect cell division, cell differentiation, and stress tolerance, among other processes, and are particularly important in modulating meristem activity and morphogenesis^37^. The key role of *CKX* in some of these processes has been illustrated by several functional studies^43,44^. Our data show an opposed accumulation pattern of CK and ABA in flower buds of peach. Consistently, CK level increases during bud dormancy release in grapevine^45^ and ABA and CK act antagonistically in the regulation of bud break in *Rosa hybrida* and Japanese pear^9,46^. Since *PpeCKX*-like is not repressed at late stages of bud development, the sharp accumulation of CKs after dormancy release in early and late flowering cultivars seems to be due to the activation of the CK biosynthetic pathway instead of CK catabolism regulation, arguing for alternative regulatory targets of *PpeDAM6* in leaves and buds.

GAs are widely considered as key regulators of bud dormancy. In fact, GA content decreases at the dormancy induction stage and increases during dormancy release in Japanese apricot^47^, pear^48^, and grapevine^49^, among others species. In sweet cherry, exogenous treatments with GA_4_ have been proposed to release dormancy of flower buds through the regulation of H_2_O_2_ content, coincidently with changes in the antioxidant defense system^50^. In *Populus*, dormancy release is associated with a restoration of plasmodesmata channels by GA_4_-induced β-1,3-glucanase expression^51^. Interestingly, GA and ABA are reciprocally regulating each other content^37^. In our study, GA levels were statistically similar comparing transgenic lines to wild-type “CV,” although GA biosynthesis, catabolism, and signaling genes are markedly different. An exogenous GA treatment increased growth in both control and 35S::*PpeDAM6* plants; however, its effect on transgenic plants was noticeably higher, leading to plants with statistically similar height. In the present study, *GA20OX2-*like and *GAST-*like genes are differentially expressed in transgenic plants but also during floral bud development in peach, suggesting they are bona fide candidate targets of *PpeDAM6*. In close agreement with our data, *PpyGAST1* and *PpyGA20OX2* gene expression increase during dormancy release in *pear*^52^. The GAST family is widely distributed among plant species and plays central roles in multiple aspects of plant growth and development, although their functions have not been completely elucidated. Members of this family have been related to flowering time control in *Arabidopsis* and Petunia^53,54^. Interestingly, the *GAST*-like GA-inducible genes *GASA4* and *GASA6* are also upregulated by auxin and CK and downregulated by ABA, JA, and salicylic acid in *Arabidopsis*^54^. In fact, *GASA6* plays a role as an integrator of GA and ABA signaling, resulting in the regulation of seed germination through the promotion of cell elongation^55^.

In addition, ABA content is known to gradually decrease during dormancy progression in different perennial species^20,56,57^. This decrease has been related to the changing expression of several biosynthesis and catabolism genes (e.g., *ZEP*-like and *NCED*-like), paralleling the behavior of dormant seeds^57,58^. Functional evidences on the participation of ABA response in growth cessation, dormancy, and cell communication have been obtained in the *Populus* model^59,60^, supporting a central role of ABA in bud dormancy processes. Our data agree with this idea and the relevant contribution of the zeaxanthin to violaxanthin conversion step, determined by VDE and ZEP activities, in *PpDAM6-*mediated regulation of ABA synthesis.

**Fig. 9:**
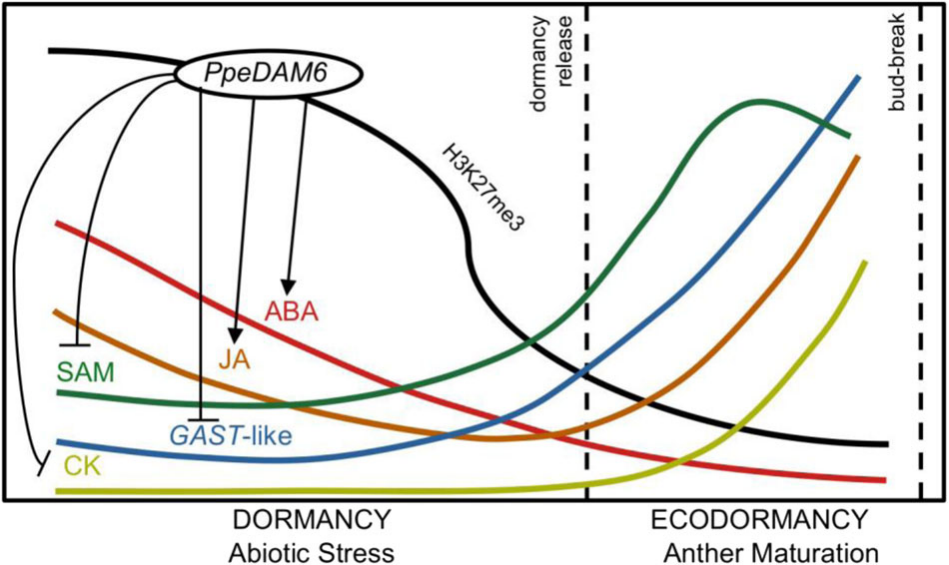
Schematic changes during flower bud development in peach. Abscisic acid (ABA), jasmonic acid (JA), citokinin (CK), and gene expression changes (PpeDAM6, GAST-like, and SAM genes). Dormancy release and bud break are labeled with dashed lines. PpeDAM6 effect on hormone accumulation and gene expression is labeled with black arrows (activation) and perpendicular bars (inhibition)

According to our data, *PpeDAM6* overexpression impinges on plant growth and development by modulating hormone contents. Results shown in this work support a model of *PpeDAM6* participation on floral bud development processes as follows. During winter dormancy, the activation of JA and ABA synthesis pathways protect the dormant floral bud against abiotic stresses, mostly low temperature and desiccation, providing a link of *PpeDAM6-*associated hormone modifications with stress adaptations. Once chilling requirements are fulfilled, *PpeDAM6* is repressed concomitantly with H3K27me3 modification on a short genomic region enriched in GA-repeat motifs. Results are coherent with BPC proteins recruiting PRC2 complexes on this region, and mediating H3K27me3 enrichment and *PpeDAM6* gene silencing. A gradual increase in *PpeGA20OX2*-like and *GAST*-like gene expression paralleling *PpeDAM6* silencing along floral bud development could modulate particular GA levels and response, contributing thus to dormancy release (Fig. 9). On the other side, a late increase in JA and CK could respectively enable anther maturation and promote cell division and differentiation in developing cells, favoring growth resumption. In conclusion, these data support a role of *PpeDAM6* as a growth repressor by modifying the hormone content.

More relevantly, *PpeDAM6* satisfies certain conditions required to be considered as the molecular basis of a physiological calendar: *PpeDAM6* is regulated by the cumulative accumulation of chilling in a genotype-dependent manner, is affected by sequential known epigenetic events, and alters key hormonal and developmental pathways in concerted fashion to modulate cell growth and tolerance to abiotic stresses.

## Materials and methods

### Plant material

Peach trees (*Prunus persica* (L.) Batsch) required in this study were grown under field conditions at Instituto Valenciano de Investigaciones Agrarias facilities. For floral bud expression analysis, the cultivar “Red Candem” (early; chilling requirement <300 chilling hours) was harvested on November 20, November 30, December 14, December 21, and January 4, whereas “Crimson Baby” (late; 400–500 chilling hours) was harvested on November 30, December 14, December 21, January 4, and January 19, during autumn–winter 2015–2016. To evaluate the dormancy status, ten budsticks from three different trees with no less than six flower buds were placed with their basal end in water in a chamber set at 24°C 12 h:12 h light:dark cycle. Routinely, the base of budsticks was cut and the water replaced with fresh one. Dormancy release was considered when more than 50% of floral buds showed at least the green tip of the sepals after 14 days. For tissue expression analysis, samples were obtained from buds (collected on January), leaves, embryos, flower parts, and fruit tissues. All plant materials used in this study are listed in Supplementary Table S4.

### Nucleic acids isolation and qRT-PCR

Leaf DNA extraction was performed according to Doyle and Doyle^61^. For RNA isolation from peach buds, 100 mg of powdered buds were extracted using RNeasy Plant Mini Kit (Qiagen), adding 1% (w:v) polyvinylpyrrolidone (PVP-40) to the kit extraction buffer before use. Leaf plum RNA extraction was performed according to Gambino et al.^62^. qRT-PCR was performed according to Lloret et al.^63^. Relative expression was measured using a relative standard curve and three biological replicates, each one with two technical replicates. All the primers used in this study are listed in Supplementary Table S5.

### Analysis of protein-DNA interaction by Y1H system

A *PpeDAM6* genomic region was divided into two fragments: Reg1 (–316 to +181 relative to the translation initiation codon), and Reg2 (+182 to +575). Reporter vectors were integrated into the genome of *Saccharomyces cerevisiae* strain Y1HGold following the Yeastmaker yeast Transformation System 2 (Takara-Bio) to create Y1H bait strains. Two microgram of total RNA obtained from a mix of dormant and non-dormant flower buds was reverse transcribed to generate the library by recombining the cDNAs with the pGADT7-rec linearized vector. The Y1H screening assay was performed following the Matchmaker Gold Yeast One-Hybrid Library Screening System (Takara-Bio) in minimal medium without leucine and supplemented with 200 ng/ml of Aureobasidin A (AbA).

To determine the DNA-binding specificity of Pwe, we used yeast strains containing the reporter vector with seven different fragments derived from pABAi-Reg2. In addition, the whole coding region of *PpeBPC3* (Prupe.8G082900) was PCR-amplified from cDNA of non-dormant floral buds and cloned into pGADT7 vector. Then, pGADT7-*PpeBPC1*, *pGADT7-PpeBPC2*, and pGADT7-*PpeBPC3* were introduced into Y1H bait strains with the seven different pAbAi-Reg2-derived plasmids.

### Analysis of protein interaction by Y2H system

The full coding sequences of *PpeBPC1, PpeBPC2, PpeLHP1, PpeSWN*, and *PpeSEUSS* were inserted into pGADT7 using the same procedure followed for *PpeBPC3* (see above). Subsequently, *PpeBPC1*, *PpeBPC2*, and *PpeBPC3* genes obtained from pGADT7 plasmids were cloned into pGBKT7 and introduced into yeast strain Y2HGold, using the Yeastmaker yeast Transformation System 2 (Takara-Bio). None of the bait constructions auto-activated the reported genes following the manufacturer’s recommended mediums with minor modifications in AbA concentration (125 ng/ml). The pGADT7 derived plasmids were sequentially introduced into the pGBKT7 transformed yeast strains. Two-hybrid interactions were tested in minimal medium without tryptophan, leucine, histidine, and adenine, and supplemented with AbA (125 ng/ml) and X-α-Gal (40 µg/ml).

### Phylogenetic analysis

For the phylogenetic analysis, BPC protein sequences from *Hordeum vulgare, Arabidopsis thaliana, Vitis vinifera*, and *Populus trichocarpa* were downloaded from TAIR10 and NCBI databases. We used ClustalW^64^ to perform multiple sequence alignment and Gblocks to remove poorly aligned positions and divergent regions of the alignment^65^. For phylogenetic tree construction, MEGA7^66^ was used with Maximum Likelihood method and tested using a Bootstrap with 1000 replicates. Nodes with less than 70% bootstrap support were eliminated.

### Dual luciferase assay

*PpeBPCs* obtained from pGADT7-*PpeBPCs* plasmids were subcloned into pGreenII-62sk vector under 35S promoter. The promoter and part of the structural region of *PpeDAM6* (–1869 to + 3575) was inserted into reporter pGreenII-0800luc vector driving firefly luciferase (LUC) expression, leading to Pro1.LUC, Pro2.LUC, and Pro3.LUC. These vectors contain the REN under a constitutive promoter that is used as an internal reference. All recombinant plasmids were individually introduced into *Agrobacterium tumefaciens* strain C58 already transformed with pSOUP, a helper plasmid that enables binary replication of pGreenII construction. *Nicotiana benthamiana* plants grown during 6 weeks were agroinfiltrated with a mix of transformed *Agrobacterium* strains. For the inoculum, an overnight culture of confluent bacteria was resuspended in the infiltration media (10 mM MgCl_2_, 10 mM MES pH 5.6) to an OD_600_ of 0.5 (HCpro strain was resuspended to an OD_600_ of 0.1). This inoculum was infiltrated on small cuts of the abaxial side of leaves with a 1 ml syringe. After 3 days, LUC activity was measured using the dual luciferase reporter assay system (Promega) with minor modifications. Two-cm leaf discs were excised, ground, and resuspended in 300 µl of lysis buffer. Ten microliter of this crude extract was assayed in 40 µl of luciferase assay buffer, and chemiluminescence was measured using GloMax Multi Microplate Reader luminometer (Promega). Three biological replicates were employed for each combination.

### Genetic transformation of plum

Transgenic plant regeneration of European plum was performed according to Petri et al.^67^. Briefly, the hypocotyl was sliced into several cross sections (less than 1 mm), which were used for transformation with *Agrobacterium tumefaciens* strain LBA4404 carrying the binary vector pROK2-*c-myc-DAM6*. After 3 days co-cultivation on shoot regenerating medium (SRM: ¾ MS based medium with 7.5 µM thidiazuron, 0.25 µM indole butyric acid, 9.05 µM 2,4-D, and 100 µM acetosyringone), the hypocotyl slices were transferred to SRM selective medium without 2,4-D and acetosyringone, and containing timentin (600 mg/l) and kanamycin (80 mg/l) during 8 weeks. Then, regenerating explants were transferred to selective hoot growing medium, in which TDZ was replaced by 1.5 µM 6-benzylaminopurine. Surviving shoot/clusters were sub-cultured at 4-week intervals at 24 °C under a 16-h photoperiod. When shoots reached 2–3 cm long, they were separated from the cluster and transferred to rooting media^68^ supplemented with kanamycin (40 mg/l) and timentin (300 mg/l). After 5–7 weeks, rooted shoots were ready for acclimatization.

### Western blot analysis

Protein extracts were obtained from 50 μg of ground leaf boiled in Laemmli buffer during 10 min at 95 °C. Samples were resolved on sodium dodecyl sulfate-polyacrylamide gel electrophoresis on 15% resolving gel and 3.5% stacking gel^69^, before transfer onto a polyvinylidene difluoride membrane (GE Healthcare-Life sciences). Membranes were blocked in 1% of blocking solution overnight at 4 °C and then incubated with Anti-myc Tag clone 4A6 (EMD Millipore) for 1.5 h. The membranes were subsequently washed and then incubated for 1 h with anti-mouse IgG POD-secondary antibody (Roche). For chemiluminescent detection, we used BM chemiluminescence western blotting kit (Mouse/Rabbit) (Roche) following the manufacturer’s protocol.

### Histological analysis of the SAM

Apices were fixed in FAE solution (4% formaldehyde, 5% acetic acid, 50% ethanol) under vacuum for 10 min and incubated overnight at 4 °C. After dehydration in alcohol series, samples were embedded in acrylic resin (Technovit 7100; Kulzer) according to the manufacturer’s instructions. Ultrathin sections were obtained and stained with 0.05% toluidine blue^70^. Slides were observed under an optical microscope (Nikon Eclipse E600). SAM height was measured in apex median longitudinal sections from the top of the SAM to the base of the rib meristem. SAM width was the distance separating the outer borders of the peripheral zone. Measurements were performed by ImageJ software (http://rsb.info.nih.gov/ij/) using digital images from at least four apices per genotype.

### RNA-seq analysis

Total RNA was extracted as shown above. Library preparation and transcriptome sequencing by paired-end sequence using Illumina HiSeqTM 2500 were conducted by Novogene Corporation. Three biological replicates from wild-type “Claudia Verde,” 35S::*PpeDAM6* #1, and 35S::*PpeDAM6* #2 were sequenced.

Raw reads with sequenced adapters, with more than 10% of uncertain bases and more than 50% of low-quality bases were removed from the analysis. Clean reads of all samples were combined and the transcriptome was assembled de novo by Trinity^71^ and filtered by CORSET^72^. To achieve comprehensive gene functional annotation, seven databases were applied (Supplementary Table S6). Cleaned RNA-seq reads were aligned to the assembled transcriptome using Bowtie^73^ through the Trinity software. Once they were mapped, reads per gene were counted by RSEM^74^ and differential expression analysis was performed on raw counts using DESeq^75^. KEGG enrichment was assessed by KOBAS^76^. Version and parameters used in each software are listed in Supplementary Table S7.

### Measurement of phytohormones

Frozen material was ground to fine powder. Before extraction, samples were spiked with 25 µl of an internal standard mixture (containing DHJA, GA_1_-*d*_*2*_ and GA_4_-*d*_*2*_ at 1 mg/l) to correct for analyte loses. Extraction was carried out in 1 ml water for 10 min in a ball mill at room temperature using 2 mm glass beads. After extraction, homogenates were centrifuged at 10,000 rpm for 10 min at 4 °C to remove debris and supernatants recovered. The resulting solutions were partitioned twice against an equal volume of di-ethyl ether after adjusting pH to 3.0 with 30% acetic acid. The combined organic layers were evaporated under vacuum and the dry residues reconstituted in 0.5 ml of a 10% aqueous methanol solution. Extracts were filtered through 0.20 µm PTFE syringe membrane filters. Samples were analyzed by tandem LC/MS in an Acquity SDS UPLC system (Waters Corp.) coupled to a TQS triple quadrupole mass spectrometer (Micromass Ltd.) through an electrospray ionization source. Separations were carried out on a C18 column (Luna Omega Polar C18, 50 × 2.1 mm, 1.6 µm particle size, Phenomenex) using a linear gradient of acetonitrile and water, both supplemented with formic acid 0.1% (v/v), at a constant flow rate of 0.3 ml/min. Hormones were detected in negative (ABA, 263 > 153; JA, 209 > 59; OPDA, 291 > 165; JA-Ile, 322 > 130; GA_1_, 347 > 229; GA_4_, 331 > 213; GA_19_, 361 > 273) or positive (iPA, 204 > 136, iPR, 336 > 204) electrospray mode following their specific precursor-to-product ion transitions and quantitated using an external calibration curve with standards.

For GA treatment, five shoots of each transgenic line were sprayed with a solution of GA_3_ (100 mg/l, 0.05% Tween-20 pH 6–7) repeatedly once per week during 1 month. The height of the plants was measured every week.

### Statistical analysis

Statgraphics XVI.I was used to assess the statistics significance. The means of two samples were compared using non-parametric Man–Whitney *U* test and comparisons of multiple samples were evaluated by non-parametric Kruskal–Wallis test with a confidence level of 95%. Significantly different samples were labeled with asterisks or different letters.

## Supplementary information

Supplementary material

## Data Availability

RNA-seq data can be found in the National Center for Biotechnology Information (NCBI) BioProject database ID PRJNA630876.
