# Supplementary material for: Regulatory circuits involving bud dormancy factor PpeDAM6
Source: Hortic Res. 2021 Dec 1;8:261. doi: 10.1038/s41438-021-00706-9 (PMC8632999; doi:10.1038/s41438-021-00706-9)
Supplement: Supplementary file 1 — Supplementary material [file 41438_2021_706_MOESM1_ESM.pdf]

>Reg1
TTTTTCTGGACAGACCAAAACTTCAGACCGGGCTGAAACCCCGACAAAGGTAAACATTAAAAACAGAGAGGAAAAC
CCAGAGATTAATTAAATTAATTAGGCTTCTTCTTCTTCTTCTTCTTCCTCTTCTTCTTTTTGTTGTGAACTTGTGACCTAT
TTTGGTTGGTTTCTTCTTCTTCTTCTTTTTGTTGTGAACTTGTGACCTATTTTGGTTGGTGGGTTTTTTCTGGGTTTTGTT
CATTTAGATCTGGGGACCATTAAAAGGTTAAATTTTGAGAAGGAACCCAGAAAATATTGTGTAGTTTTTGAGTGTATG
AAGATAATAATATATGTGAAAAGTGGTTGGTTTGAATTTTTTTGAAGGGAAACGGTGAAAATGATGAGGGAGAAGAT
CAAGATCAAGAAGATTGACTACCTGCCAGCAAGGCAGGTGACCTTCTCAAAGAGAAGAAGAGGGCTCTTCAAGAAA
GCTGCAGAGCTATCTGTTCTGTGTGAATCTGAGGTGGCTGTCATCATCTTCTCTGCTACTGACAAGCTCTTTGATTATT
CAAGCTCAAG

>Reg2
GTACCAGCACCCACCACTAAATACCTTATAATTCTTTTGACTCTTTCCTTCCTTGCAATGATAACCAATATGTATGATC
ATGATGTCTGAAGTTTGACTTGACTTGTGTGTGTGTGTGTGTGCGAGAGAGAGAGAGAGAGAGAGAGAGAGAGAGA
GAGAGAATCTGAGATTCAGTCATTTTGGTAGAAATATATTTAGAGGAGAGGGTGCCTTTTATGGAAATTACCTGTCCT
TCCTGAAATTCCATGGTTTGAACTCTGCAAACAGACATTATTTATTGTGTCAAAGGCAAATAGAAGCTTCTAGAGAGA
GAGAGAGAGAGAAAATCTTTTAGATAGTTATACTCTATTTCAAAGGCAACTAGTTCGAGGGTTACCACCTTGTATATT
TTTTATTAAGACAACTTCTAGCTAGGTGTCGCAGTTTGAGAAACATTATATGATGATGTTCTTTTCTTGTACTGCATGC
GCAATCAATTTTGTTTACCAAGCAAAACTGTGTATATCTTTTTTGGACAAAGTGTATATAAAGAAGCTGTAATCTTAT
CCTCTCCCACCTATCACATAT

>*PpeBPC1*$_{(partial)}$
TACGACGTACCAGATTACGCTCATATGGCCATGGAGGCCAGTGAATTCCACCCAAGCAGTGGTATCAACGCAGAGTG
GCCATTATGGCCGGGGAAGCCAGCAAAGAAGAGTTTGGATGTTGTGATAAACGGGATTAATATGGATATTTCAGGTA
TTCCAATTCCCGTCTGCTCATGCACTGGAGCTTCACAACAGTGTTATAGGTGGGGCTGTGGTGGTTGGCAATCTGCTT
GTTGCACCACAAACGTATCTATGTACCCTTTGCCAATGAGTACTAAGCGACGTGGGGCGAGGATAGCTGGAAGGAAA
ATGAGTCAGGGTGCTTTCAAGAAGGTGTTGGAGAAGCTTGCAGCTGAAGGTTATAATTTTGCTAACCCAATTGATCTG
AGGGGTCATTGGGCAAGACATGGTACCAACAAGTTCGTCACAATCAGGTAGAGTTGCTATGTTGGTACCATTAATGG
GACTTCAGTTGCATTTGGTCTATTTCACCTGTATATATTTCTGTGACCTTTTGCAGAGATCTATCAGATACTTTGTAGT
CAATCAATTTTCAGTCATGACGGATGATACTTGTTTCT

>*PpeBPC2*$_{(partial)}$
TACGACGTACCAGATTACGCTCATATGGCCATGGAGGCCAGTGAATTCCACCCAAGCAGTGGTATCAACGCAGAGTG
GCCATTATGGCCGGGAGAGCGAGACATGGCATTCCTGCAGCGAGATGCCGCAATTGCAGAACGGAATACTGCCATTA
TGGAACGAGACAATGCCATTGCAAATCTTCAGTACCGGGAAAACTCCTTGAATAATGGTAATGTATCTTCTTGTCCAC
CAGGATGCCAAATTTCACGTGGGGTCAAACATATGCACCACCCGCAGCAGCATGTGCATCATCCTCCTCACATGAAT
GAAGCTTCTTATGGTACAAGGGATATGCACACGAGCGATTCCGTCCCAATGCCACCTGATGCTTCTTTGCCTACAAAG
TCACGACAGCCCAAACGACCTAGGGAACCCAAGACAATGGCACCAAATAAGAAAACCTCAAAATCTCCCAGGAAAG
TGAAGAGGGAGAGTGAGGATTTAAATAAGATGACATTTGATAAATTACACGAGTGGAAGGGCAGTCAGGATATGGG
TGGCGGAGGTGATGATGTTAACAAACACTTGGTTGTGTCAAAGTCTGATTGGAAATGTCAGGACCTGGGATTGAATC
AGGTTGCATATGATGAGTCAACCATGCCAGCACCGGTGTGCTCATGCACCGGTATCCTGAGGCAGTGCTACNAGTGG
GGGA

Figure S1. Sequences of Y1H screening.

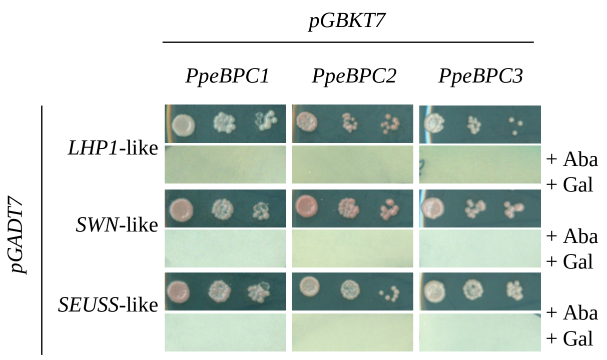

Figure S2.. Y2H analysis of protein interactions between different combinations of bait vectors (pGBKT7) containing *PpeBPC1*, *PpeBPC2* and *PpeBPC3* and prey vectors (pGADT7) containings *LHP1*-like, *SWN*-like and *SEUSS*-like,. Yeast strains were grown on a minimal medium (SD without leucine and tryptophan) and a chromogenic medium containing Aureobasidin A and X-α-Gal (+AbA +Gal).

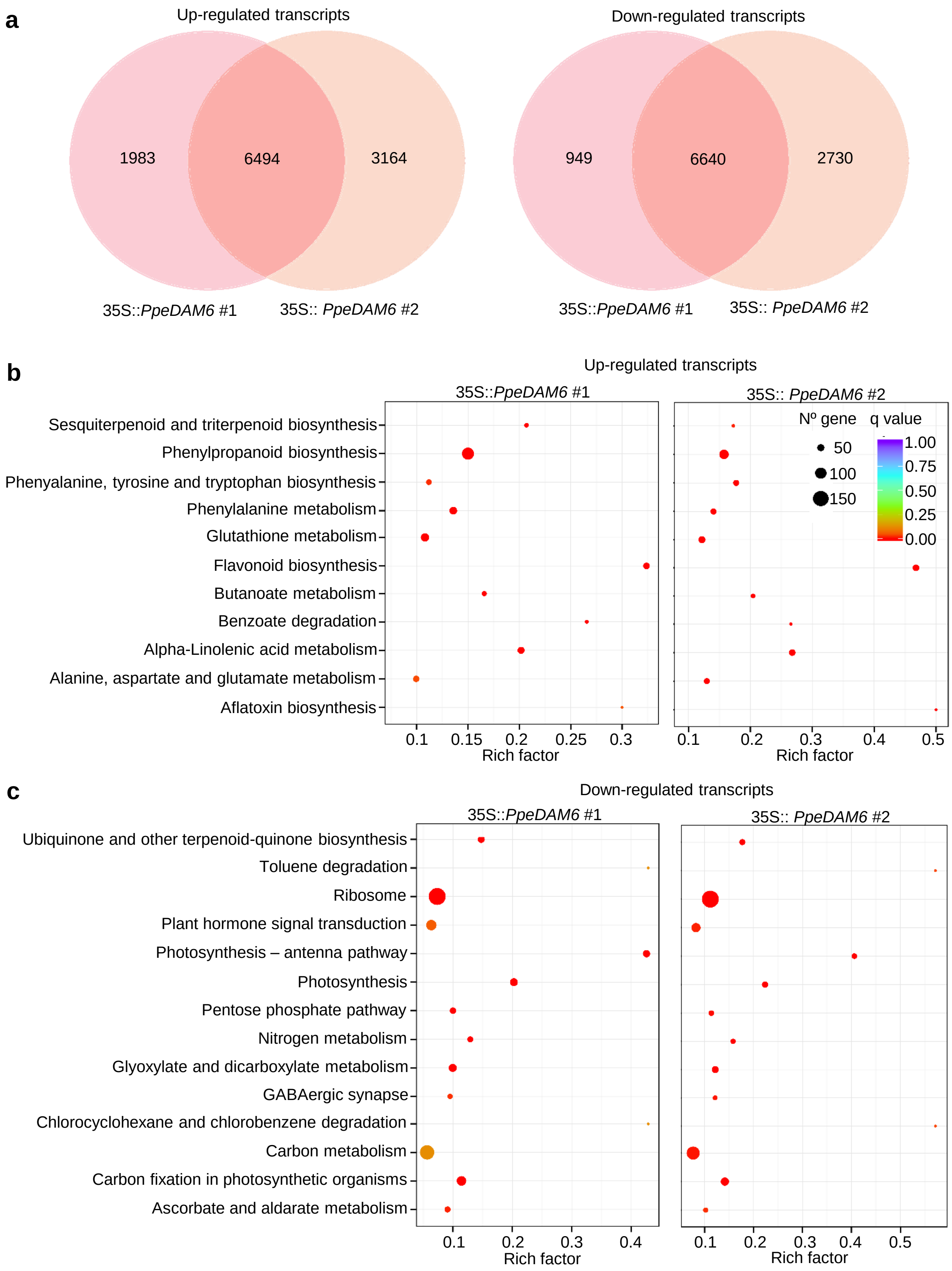

Figure S3. Transcriptomic analysis of *PpeDAM6* plum overexpressing lines. **(a)** Venn diagram of up-regulated and down-regulated transcripts of 35S::*PpeDAM6* #1 and #2 compared with control CV in leaves. **(b-c)** Significantly enriched Kyoto Encyclopedia of Genes and Genomes (KEGG) functional terms comparing 35S::*PpeDAM6* #1 and #2 vs control CV. In the scatter maps, rich factor indicates the number of enriched genes divided by the number of background genes in the corresponding pathway. Smaller q-values are closer to red colour, and the diameter of the points represent the number of Differentially Expressed Unigenes (DEUs) enriched in the corresponding function.

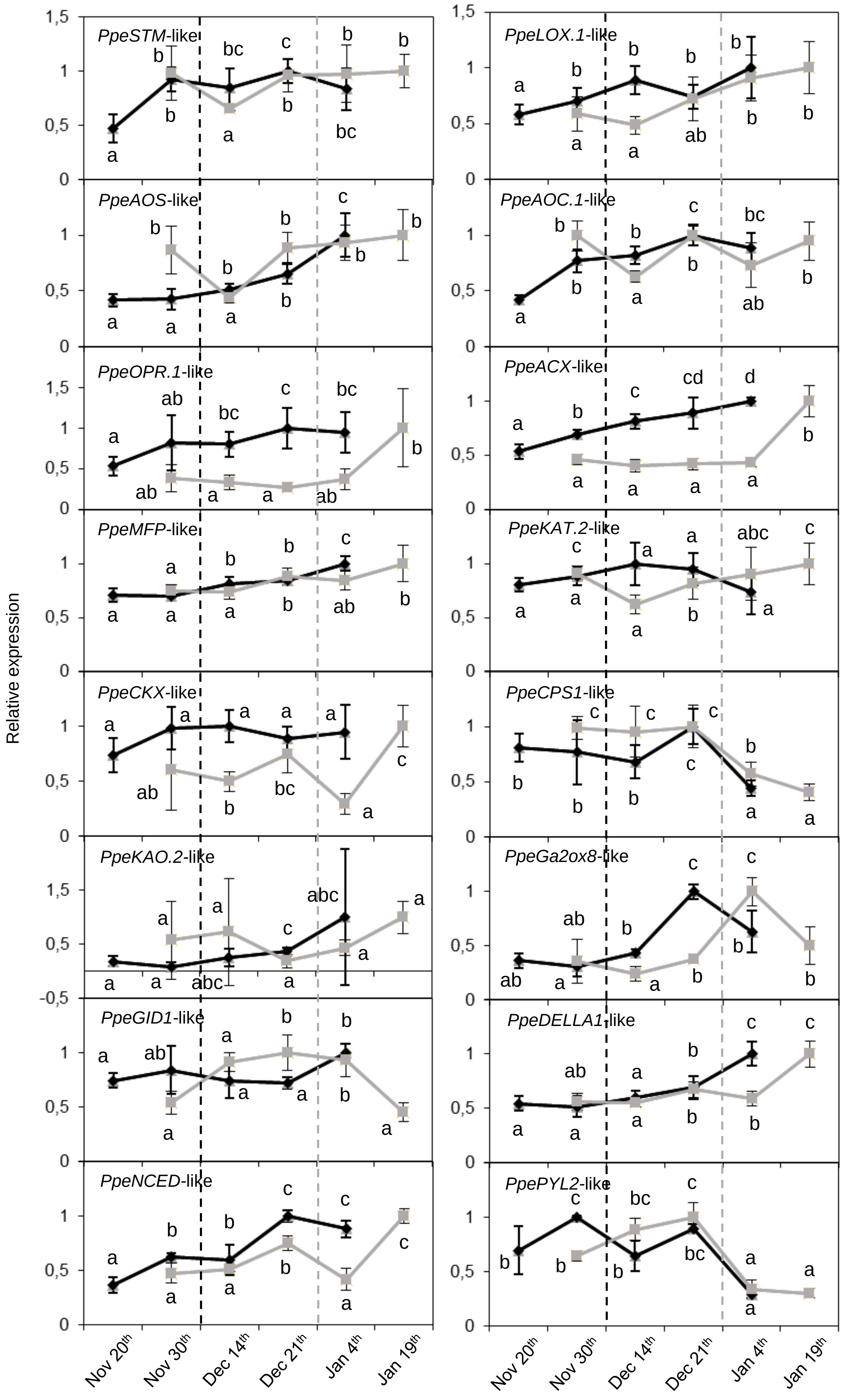

Figure S4. Relative expression of DEUs in 35S::*PpeDAM6* lines related to meristem size and hormone pathways along flower development in early (black line) and late (grey line) flowering peach cultivars. SAND-like gene was used as reference gene. Data are means from three biological samples with two technical replicates each, with error bars representing standard deviation. Different letters (a–e) indicate significant difference between samples, at a confidence level of 95%.

Table S1. Summary of sequencing data

Genotype	nº replicate	nº raw sequences	nº clean sequences
CV	1	84080676	80192132
	2	85379832	81638374
	3	80969978	76624540
35S::*PpeDAM6* #1	1	90585386	88722278
	2	94326032	91738150
	3	84776892	81478948
35S::*PpeDAM6* #2	1	107235792	104284730
	2	97445734	95165920
	3	90599952	88721900

Table S2. Summary of transcriptome assembly

Transcriptome assembly	nº unigenes	Mean length	nº unigenes >1kb
	187901	1154	75449

Table S3. Identified DEUs associated with various aspects of hormone homeostasis and response

Unigene	Gene Length (bp)	DAM2vsCV			DAM1vsCV			Hormone	Functional description	RT Abbreviature	Peach model
		Reg.	log2ratio	q value	Reg.	log2ratio	q value				
Cluster-22554.126056	987	DOWN	-1,9792	0,0078623	DOWN	-1,6712	0,037699	Abscisic acid	Abscisic acid receptor PYL2	PYL2-like	Prupe.1G413500
Cluster-22554.89003	1056	DOWN	-Inf	1,41E-05	DOWN	-Inf	3,54E-05	Abscisic acid	Carotenoid 9,10(9',10')-cleavage dioxygenase	NCED-like	Prupe.2G014700
Cluster-22554.89006	1266	UP	Inf	3,126E-05	UP	Inf	6,092E-07	Abscisic acid	Carotenoid 9,10(9',10')-cleavage dioxygenase	NCED-like	Prupe.2G014700
Cluster-22554.80257	2434	UP	1,053	1,402E-02	UP	1,061	3,862E-02	Abscisic acid	Lycopene β-cyclase		Prupe.7G046100
Cluster-22554.70311	2631	DOWN	-Inf	4,969E-93	DOWN	-6,672	1,622E-56	Abscisic acid	Phytoene synthase		Prupe.3G013200
Cluster-22554.132185	1206	DOWN	-2,140	1,942E-06	DOWN	-1,470	2,018E-03	Abscisic acid	violaxanthin de-epoxidase	VDE-like	Prupe.6G356100
Cluster-22554.119625	1868	UP	1,009	7,864E-03	UP	1,064	1,052E-02	Abscisic acid	Zeaxanthin epoxidase	ZEP-like	Prupe.8G167400
Cluster-22554.75800	1969	DOWN	-2,367	4,050E-06	DOWN	-1,371	1,734E-02	Abscisic acid	Zeaxanthin epoxidase		Prupe.8G046000
Cluster-22554.87201	3131	UP	2,552	5,333E-11	UP	2,463	3,721E-09	Abscisic acid	Zeaxanthin epoxidase		Prupe.7G133100
Cluster-22554.61360	1501	UP	1,280	3,526E-03	UP	0,924	3,500E-02	Cytoquinin	Adenylate isopentenyltransferase		
Cluster-22554.19990	1780	UP	2,100	4,012E-04	UP	2,257	4,600E-03	Cytoquinin	Cytokinin dehydrogenase	CKX-like	Prupe.1G373300
Cluster-22554.35929	2433	UP	1,867	1,765E-06	UP	1,863	1,838E-02	Cytoquinin	Cytokinin dehydrogenase	CKX-like	Prupe.1G373300
Cluster-22554.57997	1450	UP	1,413	1,635E-04	UP	1,034	2,900E-02	Cytoquinin	Cytokinin dehydrogenase		Prupe.1G404300
Cluster-22554.57998	2206	UP	1,693	1,098E-06	UP	1,332	1,067E-03	Cytoquinin	Cytokinin dehydrogenase		Prupe.1G404300
Cluster-22554.74702	1627	UP	1,857	1,966E-07	UP	1,435	6,515E-04	Cytoquinin	Cytokinin dehydrogenase		Prupe.1G404300
Cluster-22554.114841	1064	DOWN	-2,758	1,660E-07	DOWN	-2,228	9,098E-06	Cytoquinin	Cytokinin riboside 5'-monophosphate phosphoribohydrolase		
Cluster-22554.127113	1384	DOWN	-1,063	4,643E-02	DOWN	-1,115	4,805E-02	Cytoquinin	Cytokinin riboside 5'-monophosphate phosphoribohydrolase		
Cluster-22554.13145	1445	UP	Inf	2,371E-06	UP	Inf	4,830E-05	Cytoquinin	Cytokinin riboside 5'-monophosphate phosphoribohydrolase		
Cluster-22554.17424	2602	UP	8,155	1,947E-31	UP	8,672	3,210E-08	Cytoquinin	Cytokinin riboside 5'-monophosphate phosphoribohydrolase		
Cluster-24769.1	1028	DOWN	-3,338	1,084E-06	DOWN	-2,291	3,819E-04	Cytoquinin	Cytokinin riboside 5'-monophosphate phosphoribohydrolase		
Cluster-22554.87180	5608	UP	Inf	1,159E-69	UP	Inf	3,133E-24	Gibberellins	DELLA protein	DELLA-like	Prupe.3G162500
Cluster-13895.0	2529	DOWN	-Inf	1,843E-03	DOWN	-Inf	3,314E-03	Gibberellins	Ent-copalyl diphosphate synthase	CPS-like	Prupe.8G239900
Cluster-17541.0	1234	DOWN	-5,436	3,863E-04	DOWN	-3,008	2,947E-02	Gibberellins	Ent-kaurenoic acid oxidase	KAO2-like	Prupe.5G041400
Cluster-22554.14055	1940	DOWN	-1,784	7,805E-03	DOWN	-2,636	1,565E-04	Gibberellins	Ent-kaurenoic acid oxidase	KAO1-like	Prupe.2G109700
Cluster-22554.56226	341	DOWN	-Inf	1,512E-03	DOWN	-Inf	2,760E-03	Gibberellins	GA-STIMULATED TRANSCRIPT	GAST1-like	Prupe.4G257500
Cluster-22554.56227	1220	DOWN	-Inf	3,599E-12	DOWN	-5,093	5,247E-08	Gibberellins	GA-STIMULATED TRANSCRIPT	GAST1-like	Prupe.4G257500

Cluster-28710.6	933	DOWN	-2,239	1,656E-03	DOWN	-3,025	2,081E-04	Gibberellins	Gibberellin 20 oxidase 2	Ga20ox2-like	Prupe.2G150700
Cluster-28710.7	3015	DOWN	-3,194	4,541E-04	DOWN	-2,467	1,052E-02	Gibberellins	Gibberellin 20 oxidase 2	GA20ox2-like	Prupe.2G150700
Cluster-28710.8	2602	DOWN	-2,325	8,595E-08	DOWN	-1,566	9,916E-04	Gibberellins	Gibberellin 20 oxidase 2	GA20ox2-like	Prupe.2G150700
Cluster-28710.9	793	DOWN	-2,423	7,732E-03	DOWN	-1,973	4,994E-02	Gibberellins	Gibberellin 20 oxidase 2	GA20ox2-like	Prupe.2G150700
Cluster-22554.10017	1642	UP	Inf	7,154E-05	UP	Inf	5,247E-07	Gibberellins	Gibberellin 2-beta-dioxygenase 8	GA2ox8-like	Prupe.1G344000
Cluster-22554.144616	1814	UP	4,441	1,471E-03	UP	3,993	2,438E-04	Gibberellins	Gibberellin 2-beta-dioxygenase 8	GA2ox8-like	Prupe.1G344000
Cluster-22554.134519	334	UP	2,376	8,142E-04	UP	2,275	1,560E-02	Gibberellins	Gibberellin receptor GID1B	GID1b-like	Prupe.8G249800.1
Cluster-22554.134520	2438	UP	2,617	6,185E-04	UP	2,450	1,901E-05	Gibberellins	Gibberellin receptor GID1B	GID1b-like	Prupe.8G249800.1
Cluster-22554.134522	2998	UP	5,013	2,527E-11	UP	4,879	4,549E-10	Gibberellins	Gibberellin receptor GID1B	GID1b-like	Prupe.8G249800.1
Cluster-22554.134523	2349	UP	2,370	7,336E-07	UP	2,072	4,237E-05	Gibberellins	Gibberellin receptor GID1B	GID1b-like	Prupe.8G249800.1
Cluster-22554.134525	2427	UP	Inf	6,898E-41	UP	Inf	1,457E-20	Gibberellins	Gibberellin receptor GID1B	GID1b-like	Prupe.8G249800.1
Cluster-22554.134526	2974	UP	Inf	4,002E-13	UP	Inf	7,451E-09	Gibberellins	Gibberellin receptor GID1B	GID1b-like	Prupe.8G249800.1
Cluster-22554.134527	3099	UP	Inf	1,525E-02	UP	Inf	2,050E-02	Gibberellins	Gibberellin receptor GID1B	GID1b-like	Prupe.8G249800.1
Cluster-22554.134529	3063	UP	Inf	5,106E-13	UP	Inf	7,239E-07	Gibberellins	Gibberellin receptor GID1B	GID1b-like	Prupe.8G249800.1
Cluster-22554.56226	341	DOWN	-Inf	1,512E-03	DOWN	-Inf	2,760E-03	Gibberellins	Gibberellin-regulated protein	GAST1-like	Prupe.4G257500
Cluster-22554.56227	1220	DOWN	-Inf	3,599E-12	DOWN	-5,093	5,247E-08	Gibberellins	Gibberellin-regulated protein	GAST1-like	Prupe.4G257500
Cluster-22554.110632	1725	UP	1,178	6,131E-04	UP	1,049	7,651E-03	Jasmonic acid	12-oxophytodienoate reductase	OPR.2-like	Prupe.7G200800
Cluster-22554.110635	2276	UP	1,079	1,053E-02	UP	1,019	3,213E-02	Jasmonic acid	12-oxophytodienoate reductase	OPR.2-like	Prupe.7G200800
Cluster-22554.78185	858	UP	3,885	4,292E-02	UP	4,028	1,244E-02	Jasmonic acid	12-oxophytodienoate reductase		Prupe.1G548700
Cluster-22554.85674	1430	UP	1,420	2,579E-05	UP	1,496	3,616E-05	Jasmonic acid	12-oxophytodienoate reductase	OPR.1-like	Prupe.1G549000
Cluster-22554.85678	1312	UP	1,324	2,943E-04	UP	1,705	1,984E-04	Jasmonic acid	12-oxophytodienoate reductase	OPR.1-like	Prupe.1G549000
Cluster-22554.113980	1551	UP	3,091	4,496E-03	UP	3,050	6,714E-03	Jasmonic acid	3-ketoacyl-CoA thiolase 2	KAT.2-like	Prupe.8G206400
Cluster-22554.66661	1822	UP	1,230	2,649E-04	UP	1,086	4,580E-03	Jasmonic acid	3-ketoacyl-CoA thiolase 2	KAT.2-like	Prupe.8G206400
Cluster-22554.73792	1353	UP	Inf	6,177E-05	UP	Inf	3,035E-02	Jasmonic acid	3-ketoacyl-CoA thiolase 2	KAT.1-like	Prupe.1G003300
Cluster-22554.73793	546	UP	1,302	7,479E-05	UP	0,884	3,831E-02	Jasmonic acid	3-ketoacyl-CoA thiolase 2	KAT.1-like	Prupe.1G003300
Cluster-22554.73798	1621	UP	1,371	2,503E-03	UP	1,312	1,001E-02	Jasmonic acid	3-ketoacyl-CoA thiolase 2	KAT.1-like	Prupe.1G003300
Cluster-22554.73800	2024	UP	3,983	1,082E-06	UP	4,156	4,605E-03	Jasmonic acid	3-ketoacyl-CoA thiolase 2	KAT.1-like	Prupe.1G003300
Cluster-22554.74365	1899	UP	1,406	1,789E-05	UP	1,296	3,637E-04	Jasmonic acid	3-ketoacyl-CoA thiolase 2	KAT.2-like	Prupe.8G206400
Cluster-22554.74366	1892	UP	1,527	2,785E-02	UP	1,585	1,304E-02	Jasmonic acid	3-ketoacyl-CoA thiolase 2	KAT.2-like	Prupe.8G206400
Cluster-22554.74369	1904	UP	1,647	9,943E-04	UP	1,314	3,380E-02	Jasmonic acid	3-ketoacyl-CoA thiolase 2	KAT.2-like	Prupe.8G206400
Cluster-22554.74373	1395	UP	Inf	9,330E-32	UP	Inf	2,483E-27	Jasmonic acid	3-ketoacyl-CoA thiolase 2	KAT.2-like	Prupe.8G206400
Cluster-22554.93522	1946	UP	4,944	5,091E-16	UP	5,345	2,899E-19	Jasmonic acid	3-ketoacyl-CoA thiolase 2	KAT.2-like	Prupe.8G206400
Cluster-22554.73982	1958	UP	1,201	2,385E-04	UP	0,944	1,892E-02	Jasmonic acid	Acil-CoA oxidase	ACX-like	Prupe.6G279200

Cluster-22554.128014	887	UP	2,795	2,244E-18	UP	2,837	8,086E-06	Jasmonic acid	Allene oxide cyclase	AOC.2-like	Prupe.3G239900
Cluster-22554.98913	1337	UP	1,445	7,674E-06	UP	1,228	1,076E-03	Jasmonic acid	Allene oxide cyclase	AOC.1-like	Prupe.1G306100
Cluster-22554.98924	1428	UP	1,772	4,422E-02	UP	1,847	1,001E-02	Jasmonic acid	Allene oxide cyclase	AOC.1-like	Prupe.1G306100
Cluster-22554.136151	2037	UP	0,935	1,286E-02	UP	1,175	2,292E-03	Jasmonic acid	Allene oxide synthase	AOS-like	Prupe.1G386300
Cluster-22554.55584	350	UP	1,460	2,573E-04	UP	1,273	5,831E-03	Jasmonic acid	Allene oxide synthase	AOS-like	Prupe.1G386300
Cluster-20219.0	1335	DOWN	-3,588	9,851E-03	DOWN	-3,841	7,534E-03	Jasmonic acid	jasmonate O-methyltransferase		Prupe.1G375700
Cluster-22554.32274	306	UP	3,431	3,273E-10	UP	3,084	1,020E-06	Jasmonic acid	jasmonate O-methyltransferase		Prupe.8G093500
Cluster-22554.37079	290	UP	3,473	9,544E-10	UP	3,086	1,538E-06	Jasmonic acid	jasmonate O-methyltransferase		Prupe.8G093600
Cluster-22554.76170	395	UP	2,986	4,209E-06	UP	2,408	3,369E-03	Jasmonic acid	jasmonate O-methyltransferase		Prupe.8G093600
Cluster-22554.79215	1510	UP	3,415	1,398E-10	UP	3,109	2,564E-10	Jasmonic acid	jasmonate O-methyltransferase		Prupe.8G093600
Cluster-22554.104348	2829	UP	1,224	4,396E-03	UP	1,384	1,578E-03	Jasmonic acid	Jasmonic acid-amido synthetase		Prupe.2G184100
Cluster-22554.86979	1779	DOWN	-1,522	1,855E-04	DOWN	-1,459	2,622E-04	Jasmonic acid	Jasmonic acid-amido synthetase		Prupe.3G233900
Cluster-22554.86982	3291	DOWN	-1,125	9,702E-03	DOWN	-1,221	2,045E-03	Jasmonic acid	Jasmonic acid-amido synthetase		Prupe.3G233900
Cluster-22554.19621	3327	UP	Inf	4,426E-05	UP	Inf	1,431E-03	Jasmonic acid	Linoleate 13S-lipoxygenase 2-1		Prupe.4G047800
Cluster-22554.19624	3609	UP	4,704	1,424E-15	UP	4,749	1,121E-16	Jasmonic acid	Linoleate 13S-lipoxygenase 2-1		Prupe.4G047800
Cluster-22554.19625	3200	UP	Inf	1,229E-07	UP	Inf	6,854E-04	Jasmonic acid	Linoleate 13S-lipoxygenase 2-1		Prupe.4G047800
Cluster-22554.19627	1374	UP	4,542	2,017E-16	UP	4,252	1,374E-08	Jasmonic acid	Linoleate 13S-lipoxygenase 2-1		Prupe.4G047800
Cluster-22554.86196	333	UP	1,402	1,452E-04	UP	1,218	4,088E-03	Jasmonic acid	Linoleate 13S-lipoxygenase 2-1	LOX1-like	Prupe.2G005300
Cluster-22554.86201	2885	UP	1,379	1,511E-05	UP	1,292	2,764E-04	Jasmonic acid	Linoleate 13S-lipoxygenase 2-1	LOX1-like	Prupe.2G005300
Cluster-22554.86206	2874	UP	2,368	9,761E-15	UP	2,352	9,905E-06	Jasmonic acid	Linoleate 13S-lipoxygenase 2-1	LOX1-like	Prupe.2G005300
Cluster-22554.86213	3147	UP	1,605	2,766E-07	UP	1,504	1,303E-05	Jasmonic acid	Linoleate 13S-lipoxygenase 2-1	LOX1-like	Prupe.2G005300
Cluster-22554.87976	3028	UP	0,873	2,145E-02	UP	1,185	1,606E-03	Jasmonic acid	Linoleate 13S-lipoxygenase 2-1		Prupe.3G039200
Cluster-22554.88979	406	UP	1,586	1,035E-04	UP	1,416	5,831E-04	Jasmonic acid	Linoleate 13S-lipoxygenase 2-1	LOX1-like	Prupe.2G005300
Cluster-22554.106998	781	DOWN	-Inf	2,376E-02	DOWN	-Inf	3,523E-02	Jasmonic acid	Linoleate 9S-lipoxygenase 1		Prupe.6G324400
Cluster-22554.27253	1050	UP	4,982	3,444E-02	UP	4,684	2,193E-02	Jasmonic acid	Linoleate 9S-lipoxygenase 1	LOX2-like	Prupe.6G324100
Cluster-22554.99449	1000	DOWN	-3,764	1,730E-02	DOWN	-4,767	9,097E-03	Jasmonic acid	Linoleate 9S-lipoxygenase 1		Prupe.6G324400
Cluster-22554.64187	2563	UP	1,150	8,892E-04	UP	0,975	1,722E-02	Jasmonic acid	Multifunctional protein	MFP-like	Prupe.7G233700
Cluster-22554.25672	1345	UP	3,133	4,091E-05	UP	3,549	1,391E-05	Jasmonic acid	Protein TIFY		
Cluster-22554.25674	1039	UP	2,652	2,595E-07	UP	2,927	1,573E-04	Jasmonic acid	Protein TIFY		
Cluster-22554.25675	1362	UP	3,188	8,718E-08	UP	3,367	4,982E-02	Jasmonic acid	Protein TIFY		
Cluster-22554.25677	1146	UP	Inf	8,078E-04	UP	Inf	1,861E-03	Jasmonic acid	Protein TIFY		
Cluster-22554.82324	570	UP	2,832	1,907E-04	UP	2,420	8,953E-03	Jasmonic acid	Protein TIFY		
Cluster-22554.82531	2183	UP	2,308	1,771E-13	UP	2,407	1,043E-12	Jasmonic acid	Protein TIFY		

Cluster-22554.82532	2076	UP	3,380	1,619E-04	UP	3,713	1,114E-12	Jasmonic acid	Protein TIFY
Cluster-22554.82533	1526	DOWN	-1,442	1,024E-05	DOWN	-1,344	2,183E-04	Jasmonic acid	Protein TIFY
Cluster-22554.82534	2349	DOWN	-7,240	1,175E-67	DOWN	-Inf	1,225E-79	Jasmonic acid	Protein TIFY
Cluster-22554.82535	2063	UP	4,438	4,715E-12	UP	5,115	7,229E-18	Jasmonic acid	Protein TIFY

Table S4. Plant material description used along the study.

Figures or Tables	Experiment	Plant material
Fig. 1a	*PpeDAM6* expression in plant tissues	Peach tissues (see Lloret et al 2017 Sci. Rep. 7, 332)
Fig. 1b<br>Fig. 1f<br>Fig. 8b<br>Fig. S4	Gene expression along bud development	Floral buds of peach cvs ‘Red Candem’ (early flowering) and ‘Crimson baby’ (late flowering)
Fig. 3a-g	*PpeDAM6* expression and protein level in plum<br>Phenotypic analysis	Leaves of ‘Claudia Verde’ (CV) and transgenic lines 35S::PpeDAM6 #1, #2 and #3
Fig. 3f-i	Shoot apex analysis in plum	Shoot apex of ‘Claudia Verde’ (CV) and transgenic lines 35S::PpeDAM6 #1 and #2
Fig. 4b-c<br>Fig. 5b-c<br>Fig. 6b-c<br>Fig. 7b-c	Hormone-related gene expression analysis and hormone content	Leaves of ‘Claudia Verde’ (CV) and transgenic lines 35S::PpeDAM6 #1, #2
Fig. 6d	GA treatment	2-3months acclimated plants of ‘Claudia Verde’ (CV) and transgenic lines 35S::PpeDAM6 #1, #2
Fig. 8a	Hormone content	Floral buds of peach cvs ‘Red Candem’ (early flowering) and ‘Crimson baby’ (late flowering)
Fig. S3<br>Table S1-S3<br>Table S6-S7	RNA-seq analysis	Leaves of ‘Claudia Verde’ (CV) and transgenic lines 35S::PpeDAM6 #1, #2

Table S5. Primers used in this study

	Forward	Reverse
**qRT-PCR**		
PpeDAM6	TACTGGACCTGCGTTTGTGGAGCC	TGTTGCAGCTGGTGGAGGTGGCAATT
PpeDAM6 transgen	TGATCTCAGAGGAGGACCTGCATAT	GAGCTTGTCAGTAGCAGAGAAGATG
PdoDAM6-like	CCAAAACTTCAGACCGGGCTGAAA	GAGTAAACTTTCTTCCTTGTCCACTTC
PpeDAM6 + *PdoDAM6*-like	TACTGGACCTGCGTTTGTGGAGCC	TGTTGCAGCTGGTGGAGGTGGCAATT
Tubulin-like	CAGATGCCCAGTGATGCCTCAG	TGCTTGCCTGATCCAGTCTCAC
SAND-like	TCGTGGGTACCAGGAAAACGACAT	CCTGCTAGCTTGTGTTCATCTCCA
Actin-like	CTTCTTACTGAGGCACCCCTGAAT	AGCATAGAGGGAGAGAACTGCTTG
AGL26-like	ACCACCTGAAGTCCTCCAAGATTG	GCTTCATACAAAGCAATGCCAACAC
PpeBPC1	GTGATCCCGCAGTCATGGTTAG	GCATAGTTAGGATTGGCAGGCAT
PpeBPC2	GATATGGGTGGCGGAGGTGAT	CACTTGTAGCACTGCCTCAGGA
PpeBPC3	GAAGGAACCAAATGCCCTAGTCATG	GTTCACAGCATTGTCCCGCATAT
LOX1-like	CCATCCTTCTCTCTCTTACCAAAATCCT	CAGATGATCCGAGCTCACCAGAA
AOS-like	AAATGCCTTTGATGAAATCCGTCGTG	GACAAACCGATCCGCTACAAACTC
AOC.1-like	GTGTATGAGATCAACGAGAGAGACAGAG	GTTTTGGATCAGAACACATAAGCCTGC
OPR.1-like	AGCAATCAAAAACCAAAACACACAAAAGGT	AGGTTGAGCAGCCATGTCAGAG
ACX-like	GAAGTTTCACAGCCATGACAGTACCT	TCCTCAGGCGTCAATAGATCATCAAAC
MFP-like	AGTCAATCATGTCTGAAGAAGGGAAGAAG	GCTTATCTGTCCTGTGAAGAGAGCG
KAT2-like	TGCTGCCCTGTCTGCTTCAATAT	CACAATCACTACATCGTCTCCAAAAGC
CKX-like	TACTGTATTCCAACTTTTCGGCTTTTACCA	GACATAGTCGAACTTGTGGGTGGAG
CPS1-like	CACCATGTGTCTGCTGATGTGTTT	CATTTGTCTAGCAGCTCATCGGAAG
KAO.2-like	CAGAGCAAGTGGAGATTCTTAAAAGGGA	GCAGCCTCCCGATAGCCATAC
GA20ox2-like	GTTTAGTCGATGAGGCATGCAGGA	GAGAGTTGCATGCCAAAGAACAAGT
GA2ox8-like	CGAACAATCTGGGACACCGAAAG	TACACATCATTGCTCCACGCCT
GAST1-like	AGAAGCCTTGTCTGTTTTTCTGCC	GGGTCTTCCAGTTGTTGTAGCAAG
GID1b-like	CAGCCAACAGTGCCATCTATGATAC	CATGAACCTTTGAGTCCTTCCCAC
DELLA1-like	GAGAGCAGGAGAAAGCGATTGAA	TGTATGGACGAGTCTAACGCCT
ZEP-like	GTGATACTTGAGAATGGACAGCGTTATGA	GTTTGTGCCCCAAAAATACTCGGTAC
VDE-like	ACAAGTGGCTTGAATCCTACCTTTGATG	CATCATTCCTTCCACGGTAGTACACAA
NCED-like	AGGAAGCAGCGTTATGTGTATGGA	TAGCCTCAGAACCAAATCTGCCA
PYL2-like	GAGAGCCTCATCAAAACCTACCACA	AACTCAGTAACCGAAGTAACCGACC
CLV1-like	CACTTGAACATCTCCAACAACGTCTTCAG	TGCTCCAAGCTCTGCATCTCCGAG
STM-like	CACTATCACCGTCTCTTGGCTTCCTA	TACTCATCATTTGACCTATTGACGCTGCT
AGO10-like	CCTGGTTTTGGACAAGTTGGGATAAAGT	CATCATAAGCAGGCAGTCTCATTCCT
**Two-hybrid cloning**		
PpeBPC1	ACAGGATCCTCATGGATGATGATGCATTGAACA	AATCTCGAGCTACCTGATTGTGACGAACTTGT
PpeBPC2	AAAGGATCCAGATGGATGATAGTGGGCATC	CTTCTCGAGCTACTTGATTGTGATGTAGCGATTTG
PpeBPC3	TCAGAATTCATGCACTCAGCAGATAGCA	CATGGATCCTACTTGATCGTTATGTAGCGATTTG
SWN-like	CGCCATATGAGCAAAACAGGGATGGTGTC	AATGGATCCTCAGTGAGATTGGTGTTTCTTCGCT
LHP1-like	TCCCATATGAAAGTGAAGGGAGGAGGA	ATTGGATCCTTACAATGTAGAATTGTACCGGAGATG
SEUSS-like	TATGGATCCTTATGGTACCTTCGGGGC	TATGTCGACTCAAGGGGAATGTTTCCAATCA
PpeDAM6	ACCGAATTCATGATGAGGGAGAAGATCAAG	AAAGGATCCCTAGGGAAGCCCCAGTTT
**One hybrid cloning**		
Reg1	CAAGAGCTCTTTTTCTGGACAGACCAAAAC	ACACTCGAGCTTGAGCTTGAATAATCAAAGAG
Reg2	ATAGAGCTCGTACCAGCACCCACCA	GCACTCGAGATATGTGATAGGTGGGAGAGGA
Reg2.1	ATAGAGCTCGTACCAGCACCCACCA	GTACTCGAGCACACACACACACACACAAG
Reg2.2	ATAGAGCTCGTACCAGCACCCACCA	AGGCTCGAGAATCTCAGATTCTCTCTCTCTCTC
Reg2.3	ATAGAGCTCGTACCAGCACCCACCA	TCGCTCGAGTCTCTCTCTCTCTCTCTCTAGAAG
Reg2.4	GTTGAGCTCTGTGTGTGTGCGAGAGAG	GCACTCGAGATATGTGATAGGTGGGAGAGGA
Reg2.5	GAGGAGCTCGAAGCTTCTAGAGAGAGAGAGAGA	GCACTCGAGATATGTGATAGGTGGGAGAGGA
Reg2.6	GCCGAGCTCAAAATCTTTTAGATAGTTATACTCTATTTCA	GCACTCGAGATATGTGATAGGTGGGAGAGGA
Reg2.7	CGCGAGCTCATCTGAGATTCAGTCATTTTGGTAGAAA	ACCCTCGAGAGAAGCTTCTATTTGCCTTTGACA
**Luciferase assay cloning**		
Pro.1-LUC 1st part	ATAGTCGACCCGGAGCTTCTACAATGAG	GGTGGTAACCCTCGAACTAGTTGCCT

Pro.1-LUC 2nd part	GCAACTAGTTCGAGGGTTACCACCTT	TATCCATGGCGCTTTGTACCTTTCAATAACATC
**pROK2 cloning**		
PpeDAM6 + c-myc	GCGTCTAGATGATGAGGGAGAAGATCAAGATCAAG	TATGGATCCTACAGGTCCTCCTCTGAGATCAGCTT CTGCTCGGGAAGCCCCAGTTTGAG
c-myc + PpeDAM6	CAGTCTAGATGGAGGAGCAGAAGCTGATCTC	AAAGGATCCCTAGGGAAGCCCCAGTTT

Table S6. Databases used to annotate the assembled transcriptome

**Databases**	**Number of Unigenes**	**Percentage (%)**
Annotated in NR	138945	73.94
Annotated in NT	155833	82.93
Annotated in KO	55565	29.57
Annotated in SwissProt	103133	54.88
Annotated in PFAM	90455	48.13
Annotated in GO	90657	48.24
Annotated in KOG	44446	23.65
Annotated in all Databases	25454	13.54
Annotated in at least one Database	162422	86.44
Total Unigenes	187901	100

Table S7. Version and parameters used of each software in the RNA-seq analysis

Analysis	Software	Version	Parameter	Remark
**Assembly**	Trinity	r20140413p1	min_kmer_cov:2, SS_lib_type:RF, others are by default)	-
**Hierarchical Clustering**	Corset	v1.05	-m 10	remove redundancy
**Gene Functional Annotation**	Diamond	v0.8.22	NR, Swiss-Prot: e-value = 1e-5;KOG/COG: e-value = 1e-3	NR, KOG/COG, Swiss-Prot
	KAAS	r140224	e-value = 1e-10	KEGG Annotation
	NCBI blast	v2.2.28+	e-value = 1e-5	NT Annotation
	hmmscan	HMMER 3	e-value = 0.01	Pfam Annotation
	blast2go	b2g4pipe_v2.5	e-value = 1.0E-6	GO Annotation
**Mapping and Quantification**	RSEM	v1.2.26	bowtie2 mismatch 0	mapping to Corset filtered transcriptome
**Differential Expression Analysis**	DESeq	1.10.1	padj<0.05	For sample with bio-replicate using DESeq, samples without bio-replicate using DEGSeq. EdgeR for specific conditions.
**KEGG enrichment**	KOBAS	v2.0.12	Corrected P-Value<0.05	
